# The OsABF1 transcription factor improves drought tolerance by activating the transcription of *COR413-TM1* in rice

**DOI:** 10.1093/jxb/erx260

**Published:** 2017-08-12

**Authors:** Chunyu Zhang, Cong Li, Jun Liu, Yandong Lv, Chunsheng Yu, Hongyu Li, Tao Zhao, Bin Liu

**Affiliations:** 1 Institute of Crop Sciences, Chinese Academy of Agricultural Sciences, Beijing, China; 2 College of Agricultural Sciences, Heilongjiang Bayi Agricultural University, Daqing, China

**Keywords:** bZIP, COR413-TM1, drought tolerance, OsABF1, rice, transcription factor

## Abstract

Water deprivation causes substantial losses in crop yields around the world. In this study, we show that when overexpressed in transgenic rice (*Oryza sativa*), the bZIP transcription factor OsABF1 confers distinctly different drought-tolerance phenotypes when tethered to the transcriptional activator VP16 versus the transcriptional repressor EAR. We performed chromatin immunoprecipitation sequencing (ChIP-seq) and RNA sequencing (RNA-seq) assays on transgenic rice lines and determined that OsABF1 binds to DNA sequences containing an ACGT core motif. Analysis of the overlap between the RNA-sequencing and chromatin immunoprecipitation-sequencing data identified 242 OsABF1 target genes involved in multiple aspects of the drought response. Overexpression of one of these genes, *COR413-TM1*, which encodes a putative thylakoid membrane protein, resulted in a drought-tolerance phenotype without obvious side effects. In addition, OsABF1 directly regulates the expression of the *protein phosphatase 2C* (*OsPP48* and *OsPP108*) and *bZIP* (*OsbZIP23*, *OsbZIP46*, and *OsbZIP72*) genes, thus forming a complex feedback circuit in the drought/abscisic acid signaling pathway.

## Teaser

OsABF1 mediates drought tolerance by directly up-regulating the expression of drought-associated genes, including *COR413-TM1*, and forms a complex feedback circuit in the drought/abscisic acid signaling pathway.

## Introduction

Globally, drought is the primary abiotic stress that limits crop yields. Breeders have capitalized on natural occurring genetic variation to improve crop yields under drought (Eisenstein, 2013). However, in some crops natural variations that can maximize productivity under drought stress may not exist. A better understanding of physiological and molecular responses of plant under drought stress enables us to improve plant performance by breeding and genetic engineering approaches.

A hierarchy of multiple transcription factors (TFs) mediates the plant response to water deprivation (Song *et al.*, 2016). Among them, the basic leucine zipper (bZIP) TFs, containing a basic region for DNA binding and a leucine zipper motif for dimerization, belong to one of the largest TF families in eukaryotes. Plant bZIP TFs have been classified into three groups based on their DNA-binding specificity to G-box (CACGTG) or C-box (GACGTC) elements (Izawa *et al.*, 1993). To date, at least 75 and 89 bZIP TFs have been identified in the *Arabidopsis thaliana* and rice (*Oryza sativa*) genomes, respectively, and these TFs have been further classified into 10 and 11 groups, respectively, according to sequence similarities of their basic DNA-binding region or their predicted DNA-binding preferences (Jakoby *et al.*, 2002; Nijhawan *et al.*, 2008). The bZIP TFs of the different groups play diverse and critical roles in abiotic stress responses, light signaling, flower development, pathogen defense, and seed maturation. The group A bZIP TFs in *A. thaliana* and group VI bZIP TFs in rice preferentially interact with abscisic acid (ABA)-responsive elements (ABREs), which contain the (T/G/C)ACGT(G/T)GC consensus sequence and are present in the promoter regions of ABA-inducible genes. Overexpression of these bZIPs, such as *ABRE-binding Factor 3* (*ABF3*), *OsbZIP23*, or *OsbZIP72*, enhances abiotic stress tolerance in rice (Kang *et al.*, 2002; Oh *et al.*, 2005; Xiang *et al.*, 2008; Lu *et al.*, 2009), and mutations in *OsABF1/OsbZIP12* or *OsABF2/OsbZIP46* cause a hypersensitive response to drought and/or salinity stress (Amir Hossain *et al.*, 2010*a*, 2010*b*). Interestingly, overexpression of a truncated form of OsbZIP46 with a deletion of an intrinsic transcriptional repression domain, but not the intact OsbZIP46, increase the tolerance of rice to drought/osmotic stress (Tang *et al.*, 2012). However, it remains unclear how these ABRE-binding bZIP TFs target specialized downstream genes and function differentially or co-operatively in conferring tolerance to abiotic stresses. Extensive identification of the direct target genes of each bZIP TF will help elucidate their regulatory networks and how they bind to their specific targets and fine-tune gene expression in a spatio-temporal manner.

Previously, we described the approach of using hybrid transcription factors (HTFs), which cover 1500 rice transcription factors fused with the transcription activation module VP64 (a tetrameric repeat of VP16) or the repression module 4EAR (a tetrameric repeat of EAR) (Sadowski *et al.*, 1988; Beerli *et al.*, 1998; Wysocka and Herr, 2003; Imaizumi *et al.*, 2005; Song *et al.*, 2005; Song and Galbraith, 2006; Szemenyei *et al.*, 2008; Pauwels *et al.*, 2010; Kagale and Rozwadowski, 2011), to investigate the roles of different TFs in plant growth and development (Zhao *et al.*, 2015). By screening more than 50 000 independent HTF transgenic events, we identified a bZIP transcription factor, named OsABF1, which confers distinctly different flowering time phenotypes when fused with VP64 (ABF1V) or 4EAR (ABF1E) (Zhang *et al.*, 2016). Our previous study indicated that OsABF1 acts as a suppressor of floral transition in a photoperiod-independent manner. Simultaneous knockdown of both OsABF1 and its closest homologous gene, OsbZIP40, in rice by RNA interference results in a significantly earlier flowering phenotype. In this study, we further show that the *OsABF1* RNAi and overexpression transgenic lines display distinctly different drought-tolerance phenotypes in response to osmotic stress. We performed RNA sequencing (RNA-seq) and chromatin immunoprecipitation sequencing (ChIP-seq) to determine the OsABF1 binding sequence and target genes, and to investigate its regulatory network in response to drought stress. Our results demonstrate that OsABF1-mediated up-regulation of *COR413-TM1*, an *OsABF1* target gene that encodes a putative thylakoid membrane protein, may contribute to drought tolerance in rice. Moreover, OsABF1 regulates a variety of independent plant developmental processes that form a complex feedback circuit in the drought/abscisic acid signaling pathway.

## Materials and methods

### Plant material

The *ABF1V*, *ABF1E*, *ABF1F*, and *OsABF1-RNAi* transgenic plants were described in our previous study (Zhang *et al.*, 2016). To generate the *COR413-TM1* overexpression lines, *COR413-TM1* cDNA was inserted into the pHCF vector at the *Pst*I site using the Infusion system (Clontech). To generate the *COR413-TM1-RNAi* plants, a 272-bp fragment of the *COR413-TM1* gene (from 284 to 555 bp) was inserted into the pANDA vector using the Gateway cloning system (Miki and Shimamoto, 2004). Each construct was introduced into *Agrobacterium tumefaciens* strain EHA105 and then transformed into rice cv. Kita-ake. The rice T-DNA insertion mutants *osabf1-2* and *osabf1-3* were obtained from the Salk Institute Genomic Analysis Laboratory (http://signal.salk.edu/cgi-bin/RiceGE) (Jeong *et al.*, 2006).

### Growth conditions and stress treatments

Seeds of the transgenic lines and the wild-type (WT) control were soaked in Petri dishes at 37 °C for 2 d. Uniformly germinated seeds were selected for hydroponic culture in bottomless 96-well plates in 1/10 Murashige and Skoog (MS) culture solution at 28 °C with a long-day (14 h light, 28 °C; 10 h dark, 24 °C) photoperiod. For the polyethylene glycol (PEG) osmotic treatment, 3-week-old seedlings were transferred to 20% PEG 4000 solution for the days indicated and then returned to the culture solution for recovery. For the drought treatment, the indicated rice genotypes were sown and cultured with 1/10 MS culture solution in transparent boxes for 2 weeks and then transferred to boxes containing wet soil. When plants were 4 weeks old, irrigation was stopped for the indicated number of days, and then water was added to the boxes for recovery.

To analyse the expression of *OsABF1* and *COR413-TM1* under different abiotic stresses and hormone treatments, seedlings of each genotype were cultivated under continuous light at 28 °C in a plant growth chamber. At 3 weeks old, seedlings were subjected to different abiotic treatments including: 20% PEG 4000, 200 mM NaCl, 1% H_2_O_2_, cold at 4 °C, or heat at 42 °C. Hormone treatments (0.1 mM ABA, gibberellic acid, 6-benzylaminopurine, jasmonic acid, 2,4-D, or Kinetin) were performed by addition into the culture solution and spraying on the leaves (Tang *et al.*, 2012). The latest fully expanded leaves of the plants for each treatment were harvested in a time-course and used for RNA analysis.

### ChIP assay

*ABF1V-1* and WT plants grown under continuous light (CL) were used for ChIP assays, which were performed as described previously (Zhang *et al.*, 2016). Briefly, 3 g of leaves from 4-week-old seedlings were cross-linked twice by 1% formaldehyde under vacuum for 15 min and stopped using 100 mM glycine. Then the samples were ground to powder in liquid nitrogen prior to isolating chromatin. After sonication, the chromatin complexes were incubated with anti-VP16 or anti-OsABF1 antibody (Zhang *et al.*, 2016). The precipitated DNA was recovered in water for quantitative real-time PCR (qPCR) or ChIP-seq. For ChIP-qPCR, the enrichment value was normalized to that of input DNA (% of input).

### ChIP-seq data analysis

ChIP-seq clean reads were mapped to the *O. sativa* ssp. *japonica* reference genome (*Oryza sativa* MSU 6.19), which was downloaded from the ENSEMBL site (http://www.ensembl.org/index.html) after removing adaptor and low-quality nucleotides using the Bowtie2 program with parameters -x Genome.fa-U single.fq-S sample.sam-N1 (Yates *et al.*, 2016). Only the unique map reads were used for peak identification, and the *ABF1V* transgenic line and WT peaks were obtained using the model-based analysis software MACS with parameters -t sample.bam -f BAM -g 370000000 -n sample.name -w -p 1e-5 (Zhang *et al.*, 2008). ABF1V-associated genes were defined by the following criteria: (a) the overlapped length of peaks with the 1000 bp upstream of the initiation codon and coding sequence region was larger than 50% compared with peak length; (b) the value of -10[log_10_^(*P*-value)^] was larger than 80; (c) fold enrichment (FE) was larger than 5; and (d) if a peak was shared between the *ABF1V* transgenic line and the WT, the FE ratio between the *ABF1V* line and the WT should be larger than 2. To determine the consensus DNA-binding motif, peaks in the *ABF1V* line and the WT were analysed using the motif-based sequence analysis tool MEME-ChIP (Machanick and Bailey, 2011). The candidate motifs were defined by the following criteria: (a) the size of the candidate motif was set from 5 to 30 bp; (b) a motif in a similar sequence was counted once at most; (c) the *e*-value of the candidate motif was lower than 1.0E^–005^; and (d) a candidate motif could be present on both sense and anti-sense strands.

### DNA binding assay

The qPCR-based *in vitro* protein and DNA interaction assay was performed according to a previous method with minor modifications (Meng *et al.*, 2013). Briefly, the *OsABF1* coding DNA sequence (CDS) was cloned into the pCold TF plasmid at the HindIII site and transformed into the *E. coli* host strain (BL21) for the expression of recombinant protein according to the manual (TaKaRa, Cat.# 3365, v.0708). The *E. coli* cells were collected and suspended with lysis buffer [50 mM Tris (pH 8.0), 500 mM NaCl, 1 mM PMSF, and 20 mM β-mercaptoethanol], sonicated, and centrifuged at maximum speed for 1 h at 4 °C. The supernatant was diluted with lysis buffer at the designated amount and 700 μl of diluted supernatant was mixed with 50 μl of Dynabeads His-Tag beads (Life technologies, Cat. #10104D), incubated on a rotator for 5 min at room temperature, and washed five times with 1 ml washing buffer [500 mM NaCl, 50 mM Tris (pH 8.0), and 20 mM imidazole]. The beads were suspended in 50 μl of washing buffer for later use and the amount of bound protein was measured by the Bradford method. DNA fragments containing the indicated elements or non-correlated DNA fragments were synthesized and diluted to the designated concentrations. The DNA and protein binding reaction was conducted by mixing 10 μl of DNA fragments, 10 μl of beads, and 5 μl of 5×DNA binding buffer [20% glycerol, 2.5 mM DTT, 250 mM KCl, 1 mg ml^–1^ BSA, 50 mM Tris (pH 7.5), and 5 mM MgCl_2_] in 1.5-ml microcentrifuge tubes and was incubated at room temperature for 10 min. Then the beads were washed 10 times with 2×DNA binding buffer. The DNA–protein complex was eluted with elution buffer [500 mM NaCl, 50 mM Tris (pH 8.0), and 20 mM imidazole] and diluted 100-fold prior to qPCR analysis.

### RNA-seq and data analysis

The WT plants and the *ABF1V-1* and *ABF1E-1* transgenic lines were cultivated under continuous light at 28 °C for 4 weeks in plant growth chambers. The WT drought (WT-D) plants were prepared by submerging the plants in 20% PEG solution for 4 h. Ten most-recently emerged and fully expanded leaves of each genotype were collected for RNA extraction. The sequencing library was constructed following the manufacturer’s instructions (Illumina Inc.). Paired-end sequencing libraries with an insert size of approximately 200 bp were sequenced on an Illumina HiSeq 2000 sequencer at the ANOROAD Company in Beijing. RNA-seq clean reads of three biological replicates were mapped to the *O. sativa* ssp. *japonica* reference genome after removing adaptor and low-quality nucleotides by TopHat (Trapnell *et al.*, 2009). The expression value was calculated in FPKM (fragments per kilobase of exon model per million mapped fragments) and the differentially expressed genes were further analysed by Cuffdiff (*q*<0.05) (Trapnell *et al.*, 2009, 2010). Differentially expressed genes were defined as those with fold changes ≥2, or ≤2/3.

A list of all primers used in this study is provided in Supplementary Table S1 at *JXB* online.

## Accession Numbers

Sequence data from this article can be found in the MSU Rice Genome Annotation Project (http://rice.plantbiology.msu.edu/analyses_search_locus.shtml) databases (Kawahara *et al.*, 2013), under the following accession numbers: OsABF1 (LOC_Os01g64730), COR413-TM1 (LOC_Os05g49170), HOX24 (LOC_Os02g43330), AUMO1 (LOC_Os03g05880), OsEGY3 (LOC_Os03g51920), OsPP48 (LOC_Os03g16170), OsPP108 (LOC_Os09g15670), LEA14 (LOC_Os01g12580), OsbZIP23 (LOC_Os02g52780), OsbZIP46 (LOC_ Os06g10880), and OsbZIP72 (LOC_ Os09g28310). The RNA-seq and ChIP-seq data were deposited in the Gene Expression Omnibus with accession number SRP057432.

## Results

### Overexpression of OsABF1 enhances drought tolerance in rice

**Fig. 1.**
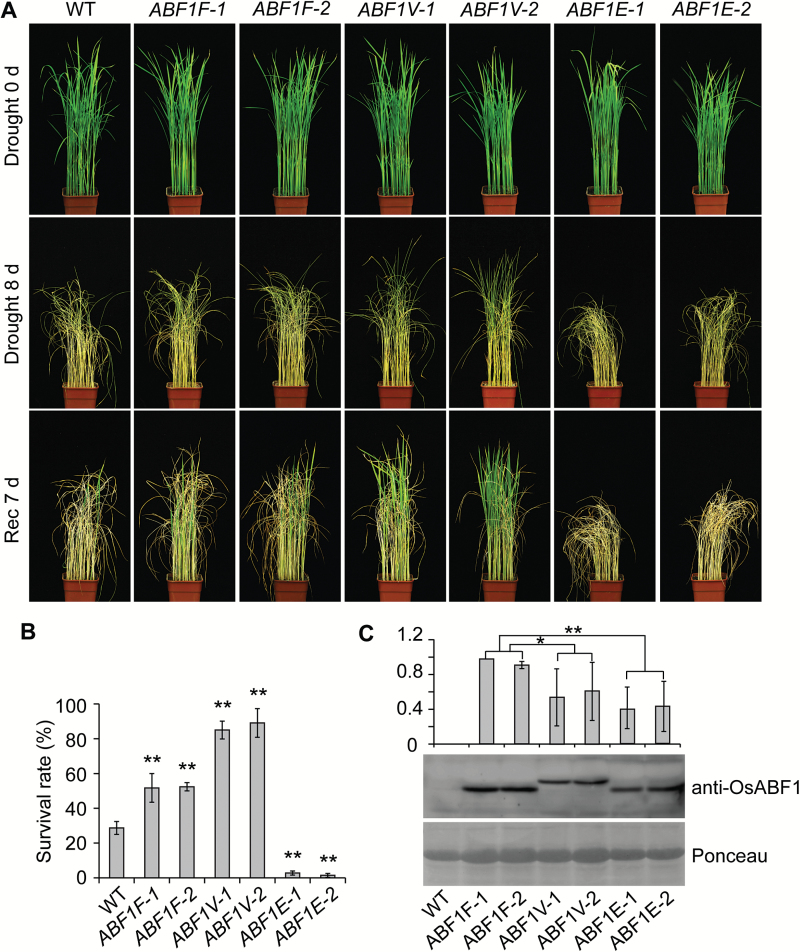
Overexpression of *ABF1F*, *ABF1V*, or *ABF1E* confers different degrees of osmotic-stress sensitivity in rice. (A) Representative images of indicated genotypes subjected to drought treatment. Plants were grown under long days (LDs, 14 h light/10 h dark) for 3 weeks before water deprivation. The images were taken before the water deprivation treatment (drought 0 d), after 8 d of water deprivation (drought 8 d), and then after water had been restored for 7 d (Rec 7 d). (B) Survival rate of each genotype subjected to water deprivation treatments as described in (A). Mean values ±SD are shown. The values of the indicated genotypes were compared to that of the WT (Student’s *t*-tests, **P<0.01, *n*=3). (C) Protein expression analysis of ABF1V, ABF1E, and ABF1F in the indicated lines. The immunoblot was probed with anti-OsABF1 antibody. The relative abundance of OsABF1 in each genotype was calculated by the formula: (OsABF1/Ponceau [each genotype])/(OsABF1/Ponceau [*ABF1F-1*]). The relative abundance of OsABF1 in *ABF1F-1* was arbitrarily set to 1. Mean values ±s.e.m. (standard error of the mean) are shown (Student’s *t*-tests, *P<0.05, **P<0.01, *n*=3).

It has been reported that two T-DNA insertion mutants in the *OsABF1* gene, *osabf1-1* (in the *O. sativa* cv. Hwayong background) and *osabf1-2* (in the *O. sativa* cv. Dongjin background; see Supplementary Fig. S1A), are more vulnerable to salinity and drought treatments, compared with wild-type rice (Amir Hossain *et al.*, 2010*b*). Here, we showed that knockdown of *OsABF1* in the *OsABF1-RNAi* transgenic lines (Zhang *et al.*, 2016) caused hypersensitivity to drought treatment, compared to the wild-type (WT) plants (*O. sativa* cv. Kita-ake; Supplementary Fig. S1B, C). Our results suggest that OsABF1 is a universal positive regulator of drought tolerance in rice. To test this hypothesis, we examined the performance of transgenic lines expressing Flag-tagged OsABF1 and OsABF1 fused to either a transcriptional activation domain or a repression domain: *Pubi:OsABF1-3Flag* (*ABF1F*), *Pubi:OsABF1-VP64* (*ABF1V*) (transcriptional activation of *OsABF1*), or *Pubi:OsABF1-4EAR* (*ABF1E*) (transcriptional repression of *OsABF1*) (see Supplementary Fig. S2A). We compared these transgenic lines with WT Kita-ake rice plants under water-deprivation conditions (Huang *et al.*, 2009). The *ABF1F* and *ABF1V* lines were significantly more tolerant to the treatment, with survival rates of about 1.8-fold and 3.0-fold higher than that of the WT, respectively. By contrast, the *ABF1E* lines were more vulnerable to drought stress than the WT (Fig. 1A, B and Supplementary Fig. S2B, C). We also used the PEG treatment to simulate drought conditions and got similar results to the water-deprivation treatment (Supplementary Fig. S3). Interestingly, although ABF1F proteins were more abundant in the *ABF1F* lines than ABF1V proteins in the *ABF1V* lines (Fig. 1C), the *ABF1F* lines were more sensitive to drought stress than the *ABF1V* lines, suggesting that the increase of the transcriptional activation activity of OsABF1 in the *ABF1V* transgenic line enhanced the performance of rice under drought stress. We also compared the OsABF1 protein level of the WT under water deprivation conditions to the *OsABF1* transgenic lines. However, due to the low sensitivity of anti-OsABF1, OsABF1 protein was not detectable in the WT, which indicated that the protein level in the OsABF1 transgenic lines was much higher than that in the WT under stressed conditions.

### Genome-wide analysis of ABF1V-associated genes by ChIP-seq

**Fig. 2.**
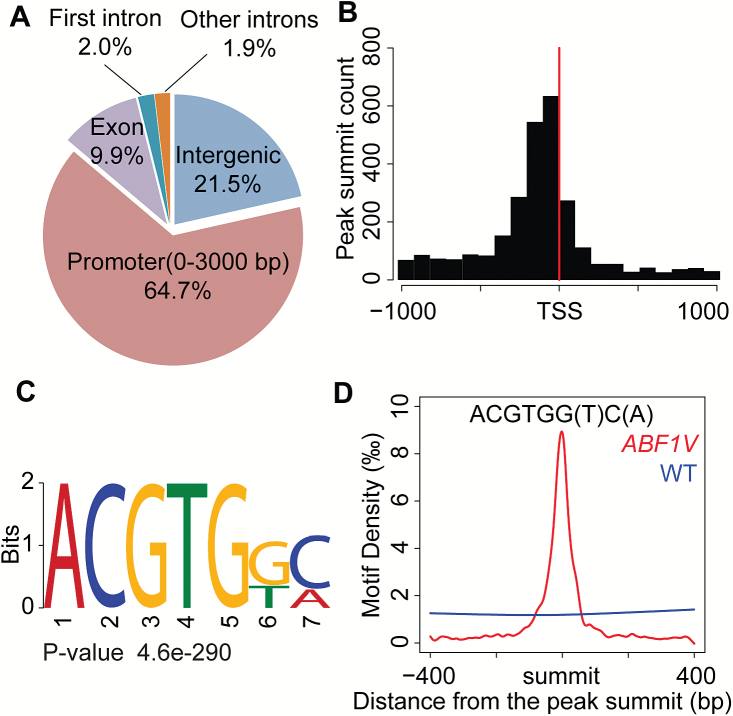
Identification of the ABF1V binding sites by analysis of the ChIP-seq data. (A) Overview of ABF1V binding peak distribution in the rice genome. (B) Enrichment of ABF1V binding peaks in the promoter region. TSS, transcriptional start site. (C) Consensus sequence identified in the ABF1V binding peaks by the MEME-ChIP program. (D) Density plot of the ACGTG(G/T)(C/A) motif around the summits of overlapping peaks in the ChIP-seq data of the *ABF1V* line drawn by the R program. The density curve of WT ChIP-seq data was used as a negative control.

To determine the direct target genes of OsABF1, we used the *ABF1V-1* transgenic line and anti-VP16 antibodies to perform chromatin immunoprecipitation and high-throughput sequencing (ChIP-seq) (see Supplementary Fig. S4). The WT Kita-ake was used as the negative control. In total, we identified 3882 genes with binding peaks localized in the promoter or CDS regions (see Supplementary Table S2). Analysis of the distribution of the sites in the rice genome demonstrated that 64.7% of the OsABF1 binding sites are located in the promoter regions, and only 35.3% are in the gene body and intergenic regions (Fig. 2A). The distance from each peak to the nearest transcription start site (TSS) was calculated and is depicted in a histogram in Fig. 2B, revealing the enrichment of association sites within 200 bp upstream of the TSSs. Furthermore, our analysis of the OsABF1 binding sequences detected an over-representation of the ACGTG(G/T)(C/A) consensus sequence (Fig. 2C). A density-plot analysis revealed that this motif was strictly enriched in the peak summits in the *ABF1V* line and the enrichment measurements returned rapidly to background levels outside ±200 bp of the peak summits. In contrast, the density was evenly distributed below 0.2% in the WT control (Fig. 2D). The ACGTG(G/T)(C/A) motif identified here is similar to the previously identified ABRE motif (T/G/C/)ACGT(G/T)GC, in that both contain an ACGT core (Ono *et al.*, 1996; Hobo *et al.*, 1999; Nijhawan *et al.*, 2008). The variation of bases surrounding the ACGT core may determine the binding specificity of individual bZIP TFs.

### Dynamic affinity of OsABF1 with its target sequences

**Fig. 3.**
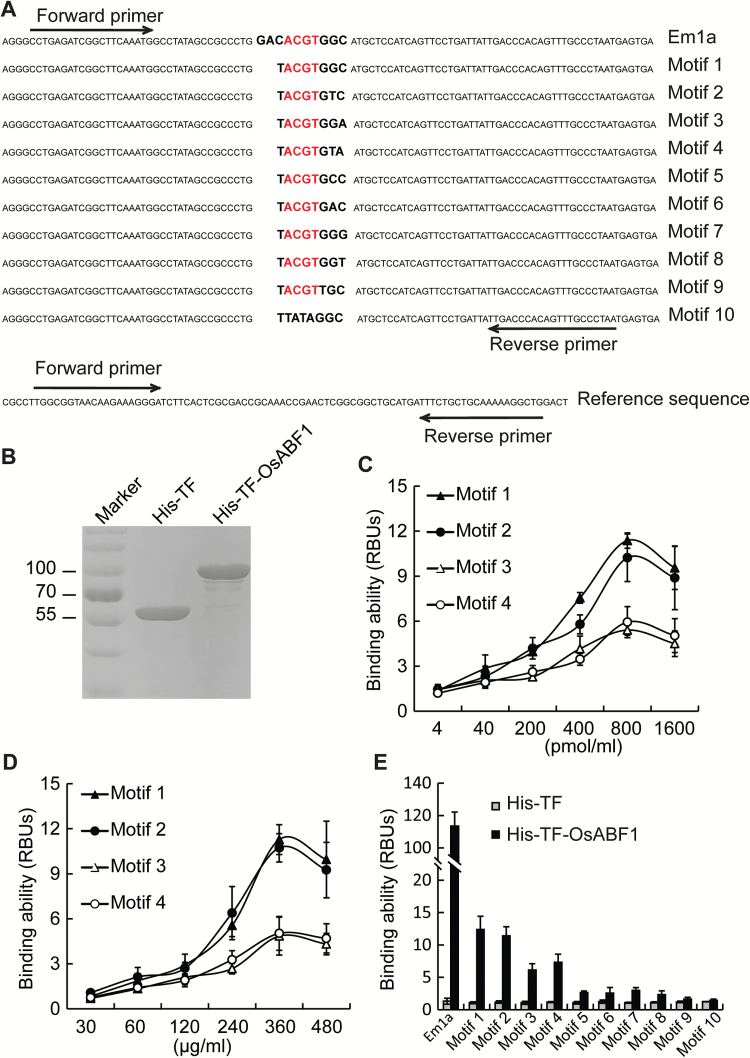
Quantitative PCR (qPCR)-based *in vitro* DNA binding assay. (A) DNA sequences containing the indicated motifs and an unrelated DNA sequence (reference sequence) used as an internal control are shown. Forward and reverse arrows indicate the primers used for qPCR. (B) The purified His-TF and His-TF-OsABF1 recombinant proteins were separated on an SDS-PAGE gel and stained with Coomassie Brilliant Blue. His-TF indicates the His-tagged trigger factor chaperone, which facilitates co-translational folding of newly expressed polypeptides. (C) Binding ability of His-TF-OsABF1 to increasing concentrations of motif 1, 2, 3, or 4. The relative binding units (RBUs) were calculated using the formula: [RBU=2^–△Ct^, △Ct=Ct^(Motif)^–Ct^(Reference)^; Ct (cycle threshold) represents the number of cycles required for the fluorescence signal to exceed the background level]. Mean values ±s.e.m. are shown (*n*=3). (D) Binding ability of each motif to increasing concentrations of His-TF-OsABF1 protein. RBUs were calculated as in (C). Mean values ±s.e.m. (standard error of the mean) are shown (*n*=3). € Binding ability of His-TF-OsABF1 to each motif. His-TF was used as a negative control.

To test if the bases immediately flanking the ACGT core affect the binding specificity of OsABF1, we analysed the binding ability of OsABF1 to different sequences containing an ACGT core via a qPCR-based protein-DNA binding assay (Meng *et al.*, 2013). DNA sequences containing the indicated motifs in conjunction with a non-specific reference sequence were synthesized commercially (Fig. 3A). The *OsABF1* CDS was cloned into the pCold TF plasmid. The His-TF (His-tagged Trigger Factor Chaperone) control protein and the His-TF-OsABF1 recombinant protein were expressed and purified from the *E. coli* host strain BL21 (Fig. 3B). To find an optimal DNA concentration for the assay, we tested the binding ability of His-TF-OsABF1 with different concentrations of motif 1 (ACGTGGC), motif 2 (ACGTGTC), motif 3 (ACGTGGA), and motif 4 (ACGTGTA) (Fig. 3C). The relative binding units (RBUs) gradually reached a peak with an increase in DNA concentration from 4 to 800 pmol ml^–1^, but then slightly decreased with a higher DNA concentration of 1600 pmol ml^–1^ (Fig. 3C). To find the appropriate protein concentration for the assay, we tested the binding ability of His-TF-OsABF1 to the indicated motif with increasing concentrations of protein (Fig. 3D). The RBUs increased gradually as the protein concentrations increased from 30 to 360 μg ml^–1^, but decreased when 480 μg ml^–1^ of protein was used. Both assays in Fig. 3C and D demonstrated that His-TF-OsABF1 has a greater ability to bind motif 1 and 2, compared with motif 3 and 4 (Factorial analysis of variance, *P*<0.001, *n*=3). When either nucleotide G or T is in the second position following the ACGT core (ACGTGGX and ACGTGTX), the OsABF1 binding ability remained the same; however, its binding ability was enhanced when C versus A is in the third position following the ACGT core (ACGTGXC vs. ACGTGXA). We investigated the binding ability of OsABF1 to 11 motif variations under the experimentally determined optimal conditions described above, using 800 pmol ml^–1^ of the DNA probe and 360 μg ml^–1^ of His-TF-OsABF1 protein (Fig. 3E). The results indicated that OsABF1 had the highest binding ability to the previously identified ABRE complex sequence named Em1a (Hobo *et al.*, 1999), which contains an ACGTGGC motif in the sense strand and an ACGTGTC motif in the antisense strand, suggesting that OsABF1 strongly prefers the site containing the ACGTG(G/T)C sequence. Moreover, the replacement of G/T with C/A (in motif 5 or 6) two nucleotides after the ACGT core significantly reduced the binding ability (Student’s *t*-test, *P*<0.001, *n*=3). The same reduction in binding ability was observed with the replacement of C/A with G/T (in motif 7 or 8) three nucleotides after the ACGT core. Furthermore, the replacement of G with T (in motif 9) immediately after the ACGT core completely stopped the interaction. Taken together, these results demonstrated that the bases immediately flanking the ACGT core are critical for the binding specificity of OsABF1 *in vitro*.

### OsABF1 regulates the transcription of genes associated with a variety of independent plant developmental processes

**Fig. 4.**
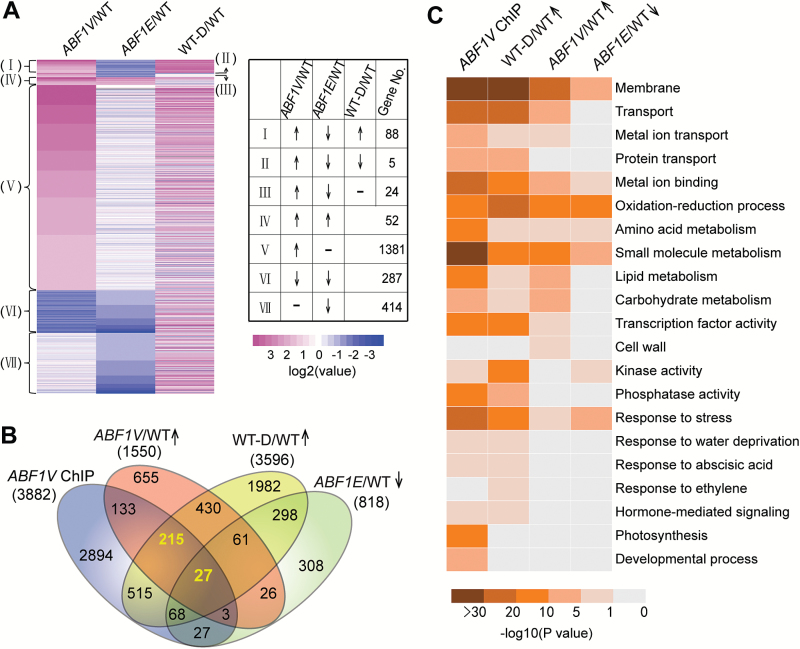
Identification of OsABF1-regulated genes by analysis of RNA-seq and ChIP-seq data. (A) Heatmap of differentially expressed genes. The *ABF1V-1* and *ABF1E-1* transgenic lines and the WT were grown under continuous light for 28 d. WT-D represents the WT sample grown under continuous light for 28 d and then treated with 20% PEG for 4 h. The color scale indicates the log-ratio calculated by normalizing the expression value of the gene in each sample to that in the WT. The differentially expressed genes were classified into seven groups from I to VII. ↑, up-regulated genes; ↓, down-regulated genes; –, unchanged genes. (B) Venn diagram showing the overlapping profiles among ABF1V-associated genes by ChIP-seq, up-regulated genes in the *ABF1V* transgenic line, up-regulated genes in the WT-D treatment, and down-regulated genes in the *ABF1E* line in comparison to that of the WT by RNA-seq. (C) Enriched functional categories in ABF1V-bound genes and in up-regulated genes in the WT-D treatment and *ABF1V* transgenic line, and down-regulated genes in the *ABF1E* transgenic line.

Next, we sought to identify genes under the regulation of *OsABF1* (or its HTFs) using the RNA-seq assay of the WT and the *ABF1V-1* and *ABF1E-1* transgenic plants grown under normal conditions, along with the WT plants treated with PEG to simulate drought conditions (WT-D) (Fig. 4 and Supplementary Fig. S5). The transcript levels from each sample were normalized to the WT expression levels. The numbers of up- and down-regulated genes were, respectively, 171 and 818 in the *ABF1E* line, 1550 and 2255 in the *ABF1V* line, and 3596 and 4089 in the WT-D (see Supplementary Tables S3, S4, and S5). The number of differentially expressed genes (DEGs) in the *ABF1E* line was much lower than that in the *ABF1V* line, suggesting that the transcriptional activation activity of OsABF1 was largely suppressed or even reversed by fusion with the 4EAR effector. To find genes associated with the abnormal phenotypes of the *OsABF1 HTF* transgenic lines, we classified the DEGs into seven groups (Fig. 4A). Among the 1550 up-regulated genes in the *ABF1V* line (Groups I–V), 117 were down-regulated in the *ABF1E* line (Groups I–III), which may account for the opposite phenotypes between the *ABF1V* and *ABF1E* lines (Fig. 1). More than 75% of these 117 genes were also up-regulated in the WT-D (Group I). In addition, cluster analysis of gene expression profiles demonstrated that the *ABF1E* line grouped together with the WT, but the *ABF1V* line was closer to the WT-D (see Supplementary Fig. S5). These results support the hypothesis that a major function of *OsABF1* is to mediate drought responses through transcriptional activation.

**Fig. 5.**
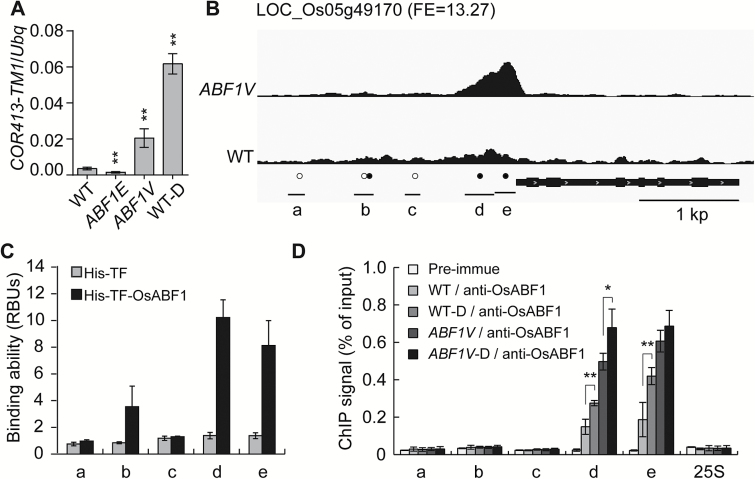
Identification of *COR413-TM1* as a direct target of OsABF1. (A) Transcriptional analysis of *COR413-TM1* in the WT, the *ABF1E* and *ABF1V* transgenic lines, and the WT-D treatment by quantitative reverse transcription PCR (qRT-PCR) with normalization to *Ubiquitin-1* (*Ubq*). Mean values ±s.e.m. (standard error of the mean) are shown (Student’s *t*-tests, **P<0.01, *n*=3). (B) ABF1V binding profile in the promoter of *COR413-TM1* in the *ABF1V* line and the WT ChIP-seq data. The bars at the bottom represent the distribution of DNA fragments containing the ACGTGG(T)C(A) motif or ACGT core, as indicated by black or white dots. (C) *In vitro* binding assay of His-TF-OsABF1 recombinant protein to the indicated DNA fragments, as shown in (B). His-TF recombinant protein was used as a negative control. (D) Verification of the OsABF1 direct binding sites in the *COR413-TM1* promoter by ChIP-qPCR analysis. ChIP samples were prepared using the *ABF1V* line and the WT plants under normal conditions or under drought stress, and precipitated with anti-OsABF1 antibodies. The pre-immune serum was used as a negative control. Results of ChIP-qPCR were quantified by normalization of the immunoprecipitation signal with the corresponding input signal. The binding to 25S rDNA was used as a negative control. The means ±SD (*n*=3) are shown.

Analyses of the overlap between the RNA-seq and ChIP-seq data revealed that 242 OsABF1 binding genes were up-regulated in both the *ABF1V* transgenic line and the WT-D (Fig. 4B). These genes were regarded as candidate target genes under the direct regulation of OsABF1 in response to drought stress. Ontology analysis revealed that genes bound by OsABF1 and genes up-regulated by drought stress are both enriched in the pathways related to membrane, transport, oxidation–reduction processes, metabolism, transcription factor activity, kinase activity, phosphatase activity, and stress response (Fig. 4C), suggesting that OsABF1 regulates the transcription of genes associated with a variety of independent plant developmental processes. We selected seven candidate target genes based on their association with these pathways to examine their transcription profiles and *in vivo* binding with OsABF1 by quantitative reverse transcription PCR (qRT-PCR) and ChIP-qPCR. The transcription of all seven candidate genes was up-regulated in the *ABF1V* line and the WT-D, but down-regulated in the *ABF1E* line (Fig. 5A and Supplementary Figs S6A–S11A). Moreover, OsABF1 physically interacted with at least one site containing the ACGTG(G/T)(C/A) motif localized within the promoter region of each of the seven candidate genes (Fig. 5B–D and Supplementary Figs S6B–D to S11B–D).

### *COR413-TM1* is a direct target of OsABF1

Membrane and stress response functional categories were highly enriched in genes bound by OsABF1, up-regulated in drought conditions, up-regulated in the *ABF1V* line, and down-regulated in the *ABF1E* line (Fig. 4C). Therefore, we further investigated one of the seven experimentally verified genes, *COR413-TM1*, which encodes a cold-inducible thylakoid membrane protein (Breton *et al.*, 2003). Its mRNA level decreased about 60% in the *ABF1E* line, increased about 4-fold in the *ABF1V* line, and increased 10-fold in the WT-D compared with the WT (Fig. 5A). ChIP-seq data showed a sharp binding peak at its promoter region, which has five sites containing an ACGTG(G/T)(C/A) motif or an ACGT core sequence (Fig. 5B). *In vitro* qPCR-based DNA binding assays indicated that recombinant His-TF-ABF1 protein efficiently binds to the b, d, and e DNA fragments of *COR413-TM1* (Fig. 5C). *In vivo* ChIP experiments using an *ABF1V* transgenic plant with anti-OsABF1 antibodies showed that ABF1V robustly binds to the d and e sites, which are immediately upstream of the TSS (Fig. 5D). Furthermore, we performed ChIP experiments using WT plants with anti-OsABF1 antibodies. Similar robust binding signals were also observed at the d and e sites, providing further validation that *COR413-TM1* is a direct target gene under the regulation of OsABF1 *in vivo*. The observation that OsABF1 binds to the b site *in vitro* (Fig. 5C) but not *in vivo* (Fig. 5D) suggested that other factors may co-operate with OsABF1 in determining the target genes in plants. Consistent with this hypothesis, the finding that *COR413-TM1* is a direct target gene of OsbZIP71, which preferentially binds to G-box (CACGTG) motifs (Liu *et al.*, 2014), further suggests that co-regulation of *COR413-TM1* expression by bZIP transcription factors is integral to the drought signaling cascade.

### OsABF1-mediated up-regulation of *COR413-TM1* in response to osmotic stress

**Fig. 6.**
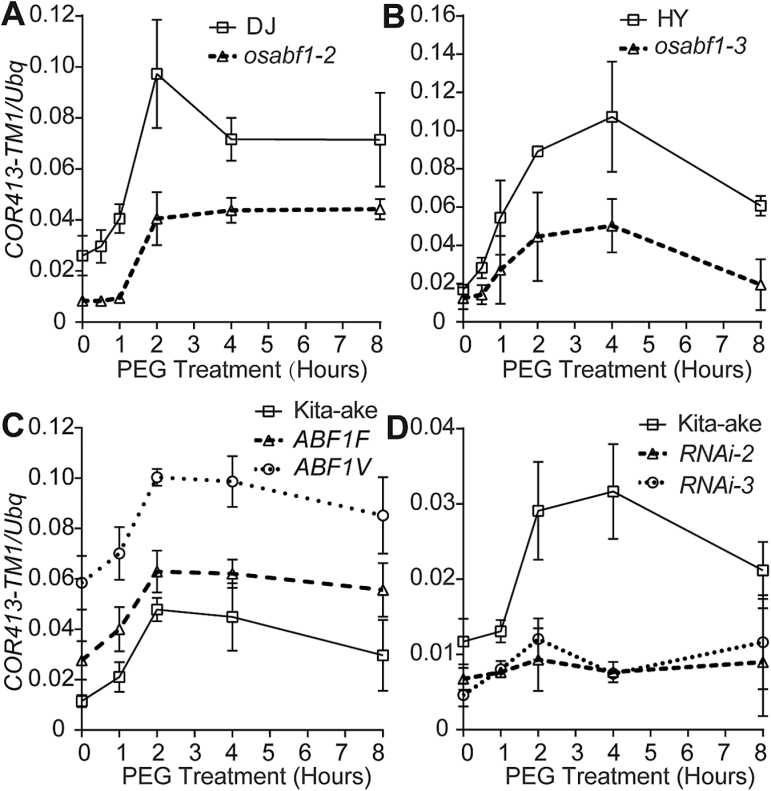
Loss of function of OsABF1 impaired induction of *COR413-TM1* expression in response to osmotic stress. (A) Dynamic changes of *COR413-TM1* mRNA levels in the *osabf1-2* mutant and wild-type Dongjin (DJ) rice. (B) Dynamic changes of *COR413-TM1* mRNA levels in the *osabf1-3* mutant and wild-type Hwayoung (HY) rice. (C) Dynamic changes of *COR413-TM1* mRNA levels in the *OsABF1F* and *OsABF1V* lines and wild-type Kita-ake rice. (D) Dynamic changes of *COR413-TM1* mRNA levels in the *OsABF1 RNAi* lines and wild-type Kita-ake rice. Mean values ±SD of three replicates are shown.

To investigate the role of OsABF1 in the regulation of *COR413-TM1* expression in response to osmotic stress, we compared the *COR413-TM1* mRNA levels in the *OsABF1* loss-of-function mutants *osabf1-2* and *osabf1-3*, and their respective WT control under PEG treatment (Fig. 6). The expression of *COR413-TM1* in the WT control was robustly induced by PEG treatment within 4 h. In contrast, the osmotic stress-induced expression of *COR413-TM1* was partially impaired in the *osabf1-2* and *osabf1-3* mutants (Fig. 6A, B), suggesting that *OsABF1* functions redundantly with other genes in activating the expression of *COR413-TM1*. Consistent with this hypothesis, we were not able to induce *COR413-TM1* mRNA levels by PEG treatment in the *OsABF1 RNAi* lines (*RNAi-2* and *RNAi-3*) (Fig. 6D), in which both *OsABF1* and its homolog *OsbZIP40* (Zhang *et al.*, 2016), are knocked out. Taken together, these results suggest that OsABF1 is required for full activation of *COR413-TM1* transcription in response to osmotic stress.

### Overexpression of *COR413-TM1* confers a drought-tolerant phenotype

**Fig. 7.**
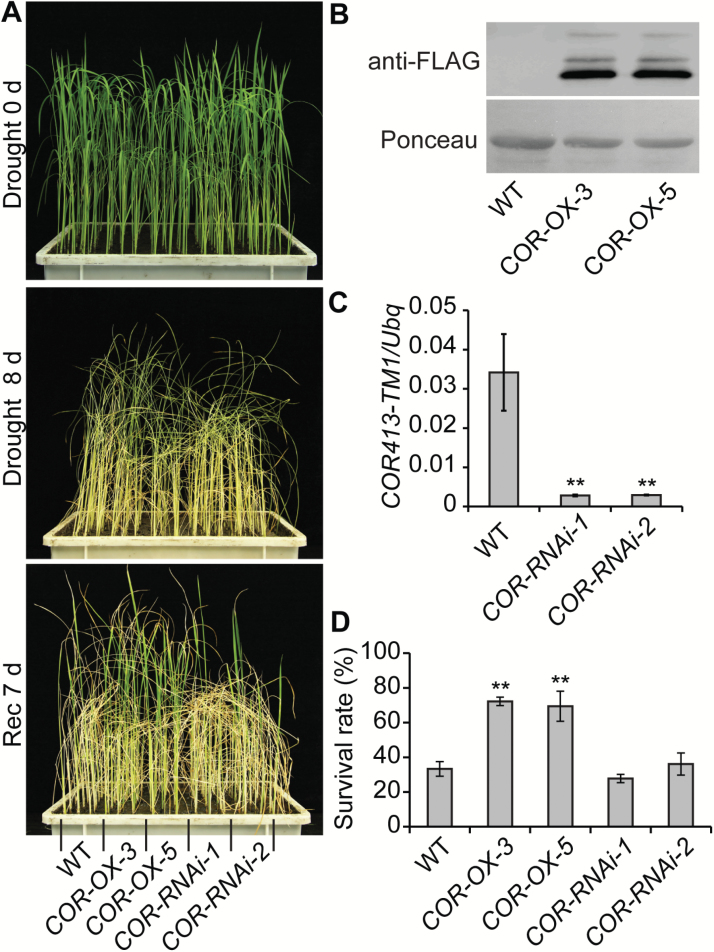
Overexpression of *COR413-TM1* confers a drought-tolerant phenotype. (A) Images of the WT, the *COR413-TM1* overexpression lines (*COR-OX-3* and *COR-OX-5*), and the RNAi lines (*COR-RNAi-1* and *COR-RNAi-2*) subjected to drought conditions. The 28-d-old seedlings were treated under drought conditions for 8 d (Drought 8 d) and then recovered by watering for 7 d (Rec 7 d). (B) Protein expression analysis of COR413-TM1-Flag in the indicated genotypes by immunoblot probed with anti-Flag antibodies. (C) Transcriptional analysis of *COR413-TM1* by qRT-PCR. Mean values ±s.e.m. (standard error of the mean) are shown (Student’s *t*-tests, **P<0.01, *n*=3). (D) Survival rate of the indicated genotypes subjected to drought treatment as in (A). Mean values ±SD are shown. The value of the indicated genotype was compared to that of the WT (Student’s *t*-tests, **P<0.01, *n*=3).

To test if *COR413-TM1* could play a role in the response to drought in rice, we obtained 28 overexpression transgenic lines (*COR-OX*s), carrying the construct *Pubi:COR413-TM1-Flag*. The osmotic stress assays demonstrated that 25 out of the 28 lines were more tolerant to both drought and PEG treatments than the WT (two representative lines are shown in Fig. 7A and Supplementary Fig. S12A). Overexpression of COR413-TM1-Flag protein in the transgenic lines was verified by western blot assays using anti-Flag antibodies (Fig. 7B). The survival rates of the transgenic lines were significantly higher than that of the WT under osmotic stress (Fig. 7D and Supplementary Fig. S12B). In addition, the transgenic lines showed no other obvious abnormal phenotypes, underscoring the potential utility of *COR413-TM1* to improve crop performance in drought conditions (Supplementary Fig. S13). The RNA interference (RNAi) approach was also applied to knockdown *COR413-TM1*. qRT-PCR analysis revealed that the expression level of *COR413-TM1* signiﬁcantly decreased in the *COR413-TM1 RNAi* lines (Fig. 7C); however, the performance of the RNAi lines under osmotic stress was similar to that of the WT (Fig. 7A, D), suggesting that functional redundancy exists between *COR413-TM1* and other genes. Taken together, these results support the hypothesis that the up-regulation of *COR413-TM1* at least partially contributes to drought tolerance mediated by OsABF1 in rice.

## Discussion

Improving drought tolerance in crops is important for sustainability in agriculture. The utilization of TFs to enhance drought tolerance in crops often results in unfavorable side effects because TFs can target thousands of downstream genes. For example, overexpression of *OsABF1* significantly improved rice performance under drought conditions but also led to a severe late-flowering phenotype. Therefore, it may be more desirable to identify the OsABF1 target genes that are specifically responsible for drought tolerance. In this study, we used *ABF1V* and *ABF1E* transgenic plants, in which the OsABF1 targets show opposite expression patterns, to identify the genes directly associated with osmotic and drought stress responses in rice. Through bioinformatics and genetic analyses, we identified a direct target gene of OsABF1, *COR413-TM1*, which encodes a putative thylakoid membrane protein specific to the plant kingdom (Breton *et al.*, 2003). In cereals and *A. thaliana*, the expression levels of several members of this gene family are regulated by cold, water stress, light, and ABA, and they may be associated with membrane structure reinforcement or environmental stress signaling (Seki *et al.*, 2002; Nijhawan *et al.*, 2008). Our data also showed that the expression of *COR413-TM1* is induced by PEG, NaCl, cold, heat, ABA, and others hormone treatments, but not by H_2_O_2_ and 6-benzylaminopurine treatments (Supplementary Fig. S14). Furthermore, we demonstrated that OsABF1 mediates drought-induced up-regulation of *COR413-TM1* (Figs 5 and 6), and overexpression of *COR413-TM1* enhanced drought tolerance in transgenic rice without other side effects (Fig. 7 and Supplementary Fig. S13), suggesting its potential utility in developing drought-tolerant crops. It is interesting to note that the drought tolerance of *COR413-TM1* was not as strong as that of the *ABF1V* line (Figs 1B and 7B), suggesting that an additive effect with other target genes may exist. *LEA14* (encoding late embryo abundant protein 14) is also a target of OsABF1 (Supplementary Table S6 and Supplementary Fig. S9), and overexpression of LEA proteins has been shown to enhance drought tolerance in transgenic rice (Xiao *et al.*, 2007; Duan and Cai, 2012). Therefore, OsABF1 appears to mediate drought tolerance by modulating the expression of multiple downstream genes, including *COR413-TM1* and *LEA14*.

**Fig. 8.**
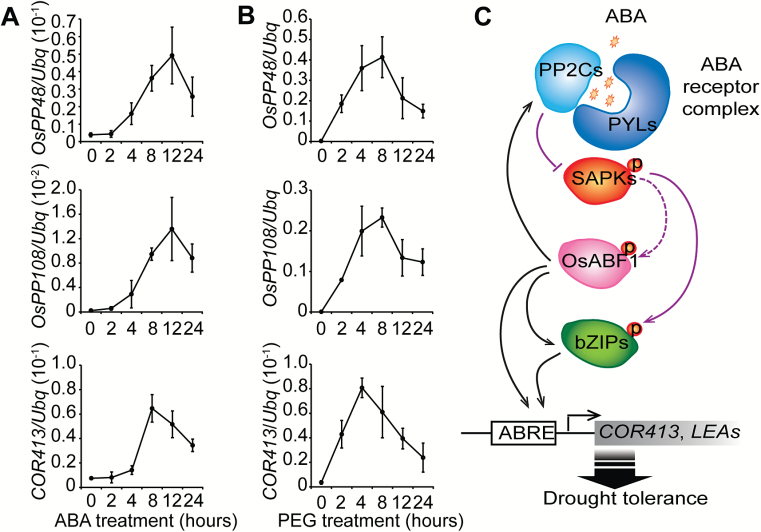
OsABF1 mediates a feedback regulation pathway in ABA signaling and osmotic stress/drought responses. (A) Dynamic changes of the mRNA levels of each gene in response to ABA treatment. (B) Dynamic changes of the mRNA levels of each gene in response to PEG treatment. (C) A working model showing the crucial role of OsABF1 in the ABA-dependent pathway in osmotic/drought stress regulation. Regulation at the transcriptional level is depicted by black arrows and at the post-transcriptional level by purple arrows. The dashed line indicates predicted regulation.

Interestingly, we found two clade-A *protein phosphatase 2C* (*PP2C*) genes, *OsPP48* and *OsPP108*, that are under the direct regulation of OsABF1 (Supplementary Table S6 and Supplementary Figs S10, S11). The PP2C and Pyrabactin resistance1 (PYR)/PYR1-like (PYL)/RACR proteins are the components of the ABA receptor discovered in *A. thaliana* (Ma *et al.*, 2009; Park *et al.*, 2009). The phosphatase activities of PP2Cs are inhibited by ABA-bound PYR/PYL/RACR, releasing the activation of the subclass III SnRK2s (SAPKs), which further phosphorylate TFs including ABFs and AREBs (Furihata *et al.*, 2006; Fujii and Zhu, 2009; Fujita *et al.*, 2009; Yoshida *et al.*, 2010). In rice, OsPP48/OsPP2C30, OsPYL/RACR5, and SAPK2 (a homolog of the SnRK2 protein kinase) were reported to form a complex to mediate ABA signaling (Kim *et al.*, 2012). Because both drought and ABA treatments increase the expression of *OsABF1*, which further up-regulates the expression of *OsPP48* and *OsPP108*, it may be that PYR/PYLs/RACR, SAPKs, PP2Cs, and OsABF1 form a negative feedback regulatory loop in the ABA signaling pathway (Fig. 8). We also found that OsABF1 directly regulates the expression of *OsbZIP23*, *OsbZIP46*, and *OsbZIP72*, which are associated with osmotic stress tolerance in rice (Hobo *et al.*, 1999; Xiang *et al.*, 2008; Lu *et al.*, 2009; Tang *et al.*, 2012) (Supplementary Fig. S15). OsbZIP23 can activate the expression of *OsPP76*/*OsPP2C49*, which revealed feedback regulation of ABA signaling (Zong *et al.*, 2016). Our finding that OsABF1 directly targets both *bZIP*s (*OsbZIP23*, *OsbZIP46*, and *OsbZIP72*) and *PP2C*s (*OsPP48* and *OsPP108*) adds further insight into the complex drought–ABA signaling network in rice (Fig. 8).

## Supplementary Data

Supplementary data are available at *JXB* online.

Fig. S1. Diagrams of T-DNA insertion positions and the performance of *OsABF1-RNAi* lines under osmotic stress.

Fig. S2. Diagram of the OsABF1 HTF constructions and the performance of *OsABF1* transgenic lines under water deprivation conditions.

Fig. S3. Performance of *OsABF1V*, *OsABF1E*, and *OsABF1F* lines under polyethylene glycol (PEG) treatment.

Fig. S4. Genome-wide distribution of ABF1V-associated sites.

Fig. S5. Heat map of RNA-seq data.

Fig. S6. Identification of *HOX24* as a direct target of OsABF1.

Fig. S7. Identification of *AUMO1* as a direct target of OsABF1.

Fig. S8. Identification of *OsEGY3* as a direct target of OsABF1.

Fig. S9. Identification of *LEA14* as a direct target of OsABF1.

Fig. S10. Identification of *OsPP48* as a direct target of OsABF1.

Fig. S11. Identification of *OsPP108* as a direct target of OsABF1.

Fig. S12. Performance of *COR413-TM1* overexpression lines under osmotic stress.

Fig. S13. Yield traits of *COR413-TM1* overexpression lines.

Fig. S14. Dynamic transcription of *OsCOR413-TM1* under abiotic stress or hormone treatments.

Fig. S15. Identification of *OsbZIP23*, *OsbZIP46*, and *OsbZIP72* as direct targets of OsABF1.

Table S1. Oligonucleotide primers used in this study.

Table S2. ABF1V-associated genes.

Table S3. Differentially expressed genes between the WT and the *ABF1V* transgenic line.

Table S4. Differentially expressed genes between the WT and the *ABF1E* transgenic line.

Table S5. Differentially expressed genes between the WT and the WT-D treatment.

Table S6. Candidate genes directly regulated by OsABF1.

## Author contributions

BL and ZT conceived this project and designed the research; CZ, CL, JL, YL, CY, and HL performed the research; BL and ZT wrote the article with contributions from all the authors.

## Supplementary Material

supplementary figuresS1-S15

Supplementary Table S1

Supplementary Table S2

Supplementary Table S3

Supplementary Table S4

Supplementary Table S5

Supplementary Table S6
