# Supplementary material for: The OsABF1 transcription factor improves drought tolerance by activating the transcription of COR413-TM1 in rice
Source: J Exp Bot. 2017 Aug 12;68(16):4695–707. doi: 10.1093/jxb/erx260 (PMC5853872; doi:10.1093/jxb/erx260)
Supplement: supplementary figuresS1-S15 [file erx260_suppl_supplementary_figures_s1_s15.pdf]

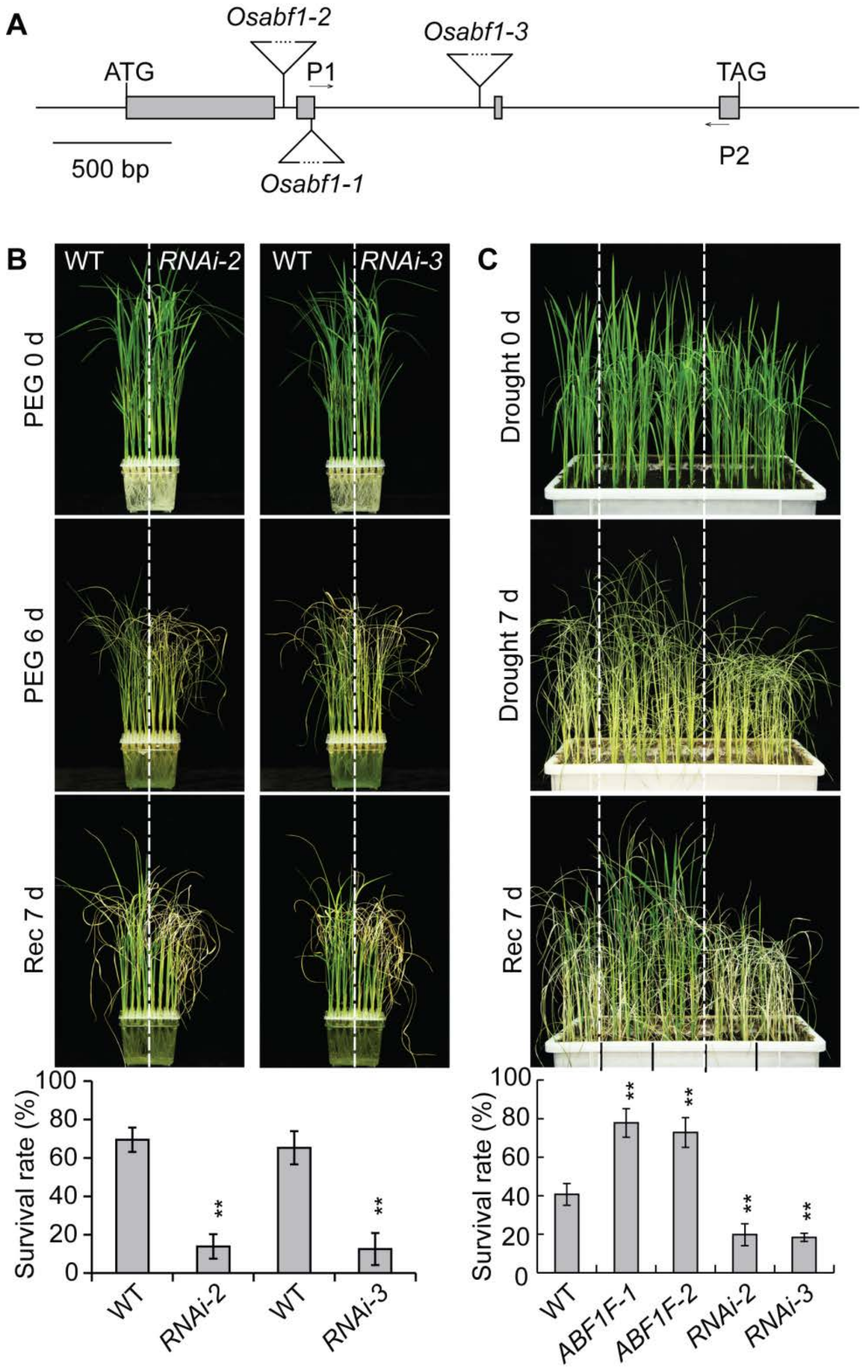

Supplemental Figure S1. Diagrams of T-DNA insertion positions and the performance of *OsABF1-RNAi* lines under osmotic stress. A. Schematic representation of the T-DNA insertion mutants in *OsABF1* gene, *osabf1-1*, *osabf1-2* and *osabf1-3*. B. Images of the WT and *OsABF1-RNAi* transgenic plants taken before 20% PEG treatment (PEG 0 d), after the roots of seedling were submerged in PEG for 6 days (PEG 6), and then after plants were restored to water for 7 days (Rec 7). C. Images of the WT, *OsABF1-RNAi* and *ABF1F* transgenic plants taken before water deprivation treatment (Drought 0 d), after water deprivation for 7 days (Drought 7 d), and then after plants were restored to water for 7 days (Rec 7 d). Survival rate with statistics after recovery. Mean values ± s.d. are shown. The values of the indicated genotypes were compared to that of the WT (Student's *t*-tests, ***p* < 0.01, n = 3). .

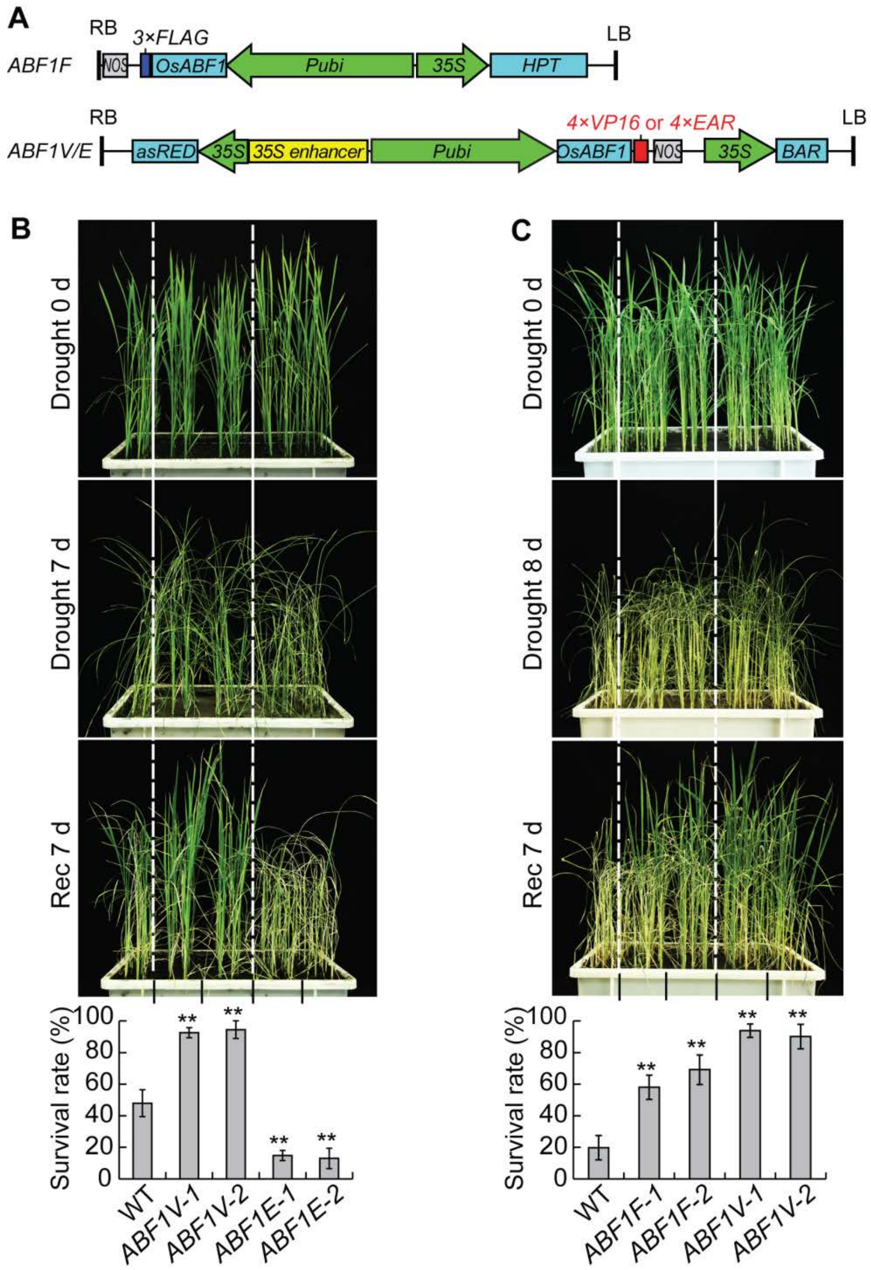

Supplemental Figure S2. Diagram of the OsABF1 HTF constructions and the performance of *OsABF1* transgenic lines under water deprivation condition. A. Diagrams of the OsABF1 HTF constructions. B. Images of the WT, *OsABF1V* and *OsABF1E* transgenic plants taken before water deprivation treatment (Drought 0 d), after water deprivation for 7 days (Drought 7 d), and then after plants were restored to water for 7 days (Rec 7 d). C. Images of the WT, *OsABF1F* and *OsABF1V* transgenic plants taken before water deprivation treatment (Drought 0 d), after water deprivation for 8 days (Drought 7 d), and then after plants were restored to water for 7 days (Rec 7 d). Survival rate with statistics after recovery. Mean values ± s.d. are shown. The values of the indicated genotypes were compared to that of the WT (Student's *t*-tests, ***p* < 0.01, n = 3).

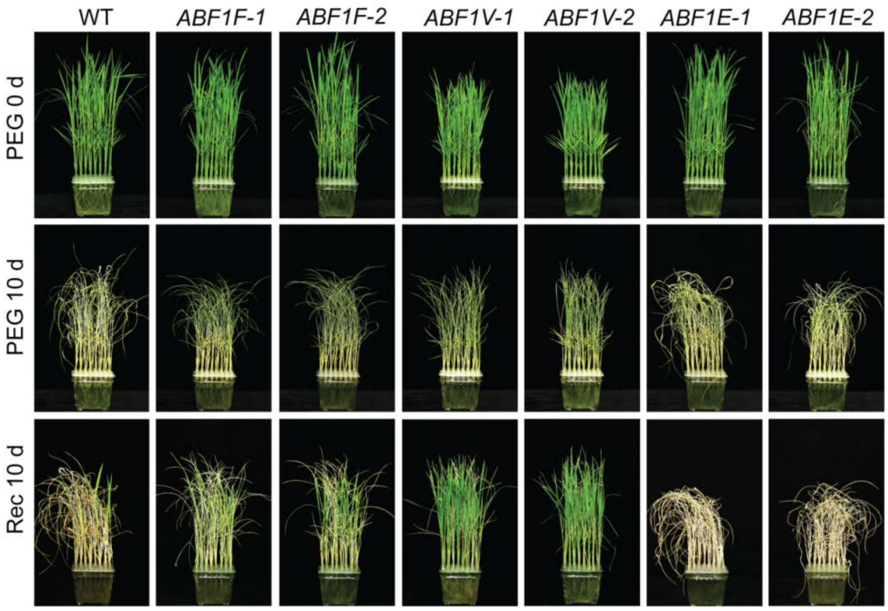

Supplemental Figure S3. Performance of OsABF1V, OsABF1E and OsABF1F lines under polyethylene glycol (PEG) treatment. Representative images of indicated genotypes subjected to PEG treatment. Plants were grown under long days (LDs, 14 hours light/10 hours dark) for three weeks before treatment with 20% PEG. The images were taken before PEG treatment (PEG 0 d), after the roots of seedling were submerged in PEG for 10 days (PEG 10) and then restored to water for 10 days (Rec 10) respectively.

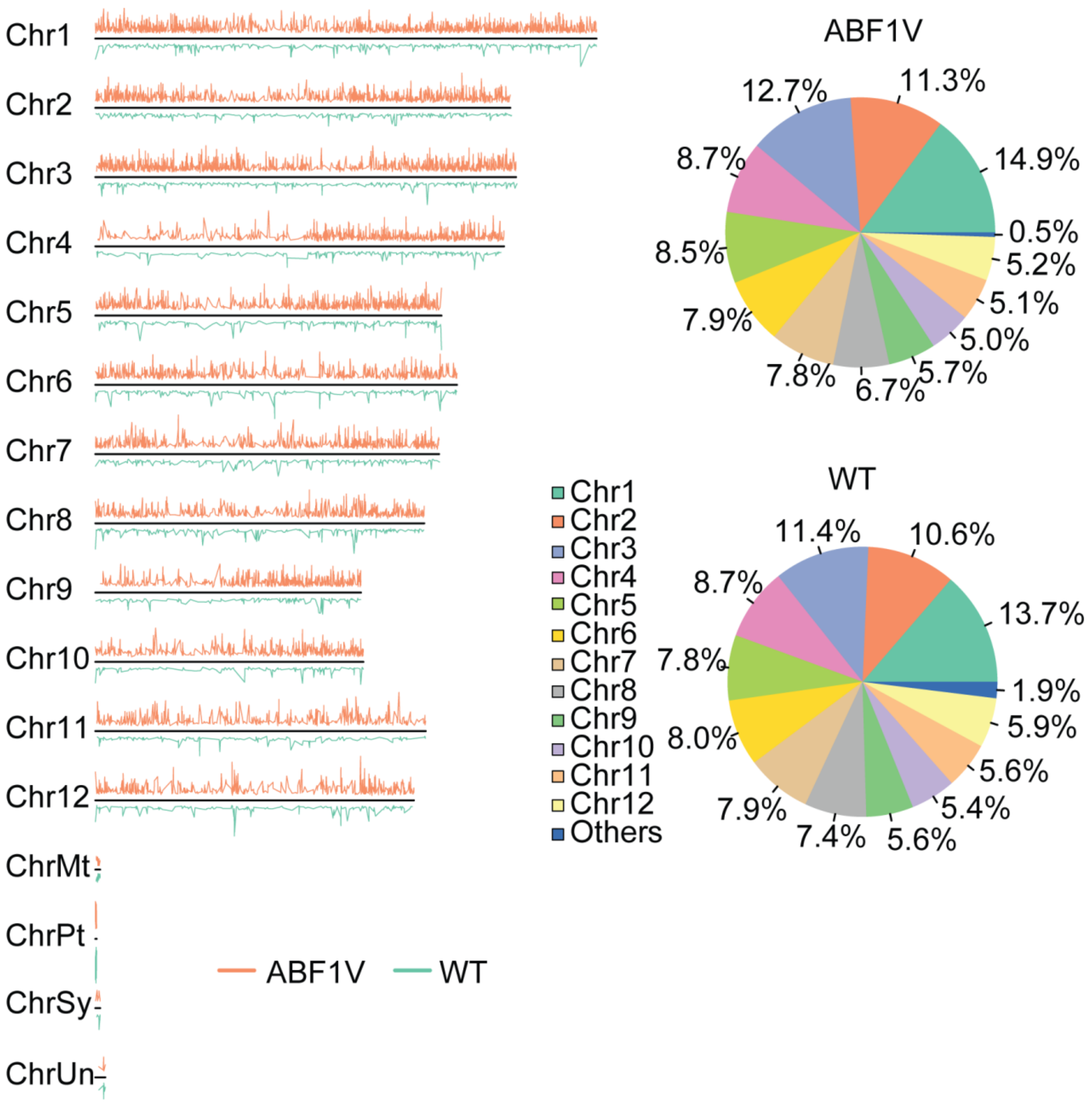

Supplemental Figure S4. Genome-wide distribution of ABF1V-associated sites. The numbers in the pie charts indicate the percentage of peaks in each chromosome.

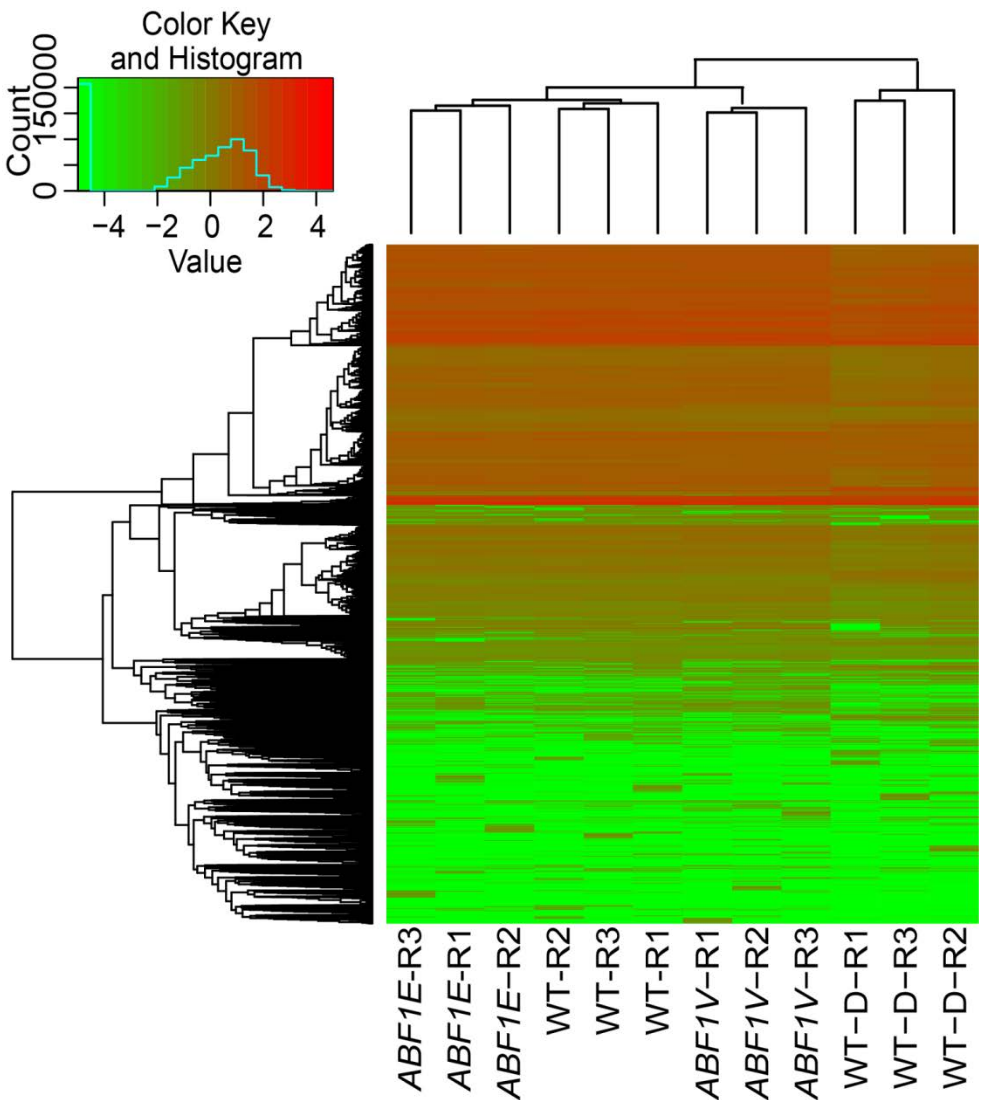

Supplemental Figure S5. Heat map of RNA-seq data.

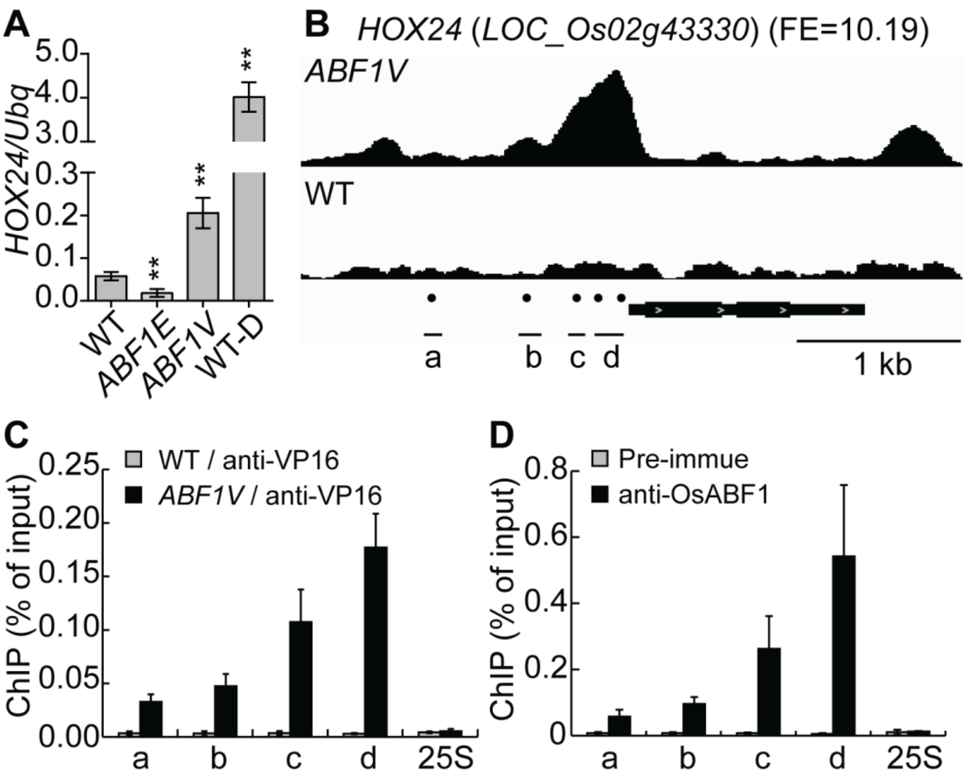

Supplemental Figure S6. Identification of *HOX24* as a direct target of ABF1V. A, Transcriptional analysis of *HOX24* in the WT, *ABF1E* and *ABF1V* transgenic lines, and the WT-D by qRT-PCR with normalization to *Ubq*. Mean values ± s.e.m. are shown (Student's *t*-tests, $**p < 0.01$, n = 3). B, ABF1V binding profile in the promoter of *HOX24* in the ChIP-seq data. The bar at the bottom represents the position of the DNA fragment containing the ACGTG(G/T)(C/A) motif which indicated with black dots. C, Verification of OsABF1V direct binding to the indicated DNA fragments as shown in B by ChIP-qPCR analysis. ChIP samples were prepared using anti-VP16 antibodies and subjected to qPCR analysis. Results of ChIP-qPCR were quantified by normalization of the immunoprecipitation signal to the corresponding input signal. The binding to 25S rDNA was used as a negative control. The means ± s.d. (n = 3) are shown. D, Verification of OsABF1 direct binding sites in the *HOX24* promoter by ChIP-qPCR analysis. ChIP samples were prepared using WT rice and precipitated with anti-OsABF1 antibodies or with the pre-immune serum as a negative control. The means ± s.d. (n = 3) are shown.

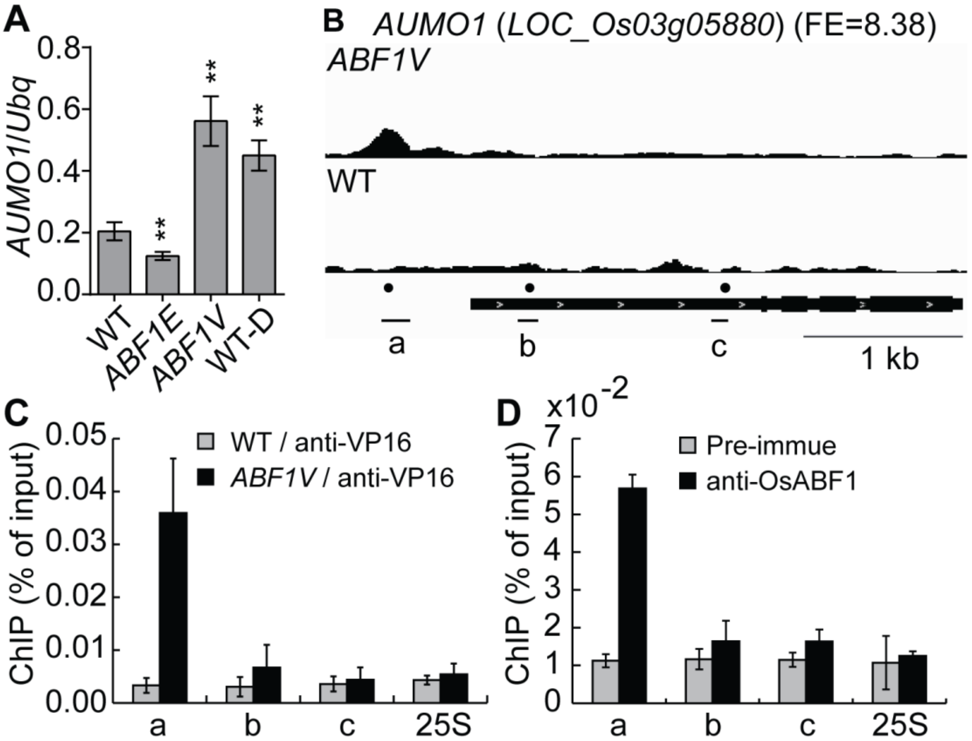

Supplemental Figure S7. Identification of *AUMO1* as a direct target of OsABF1. A, Transcriptional analysis of *AUMO1* in the WT, the *ABF1E* and *ABF1V* transgenic lines, and the WT-D by qRT-PCR with normalization to *Ubq*. Mean values ± s.e.m. are shown (Student's *t*-tests, $**p < 0.01$, n = 3). B, ABF1V binding profile in the promoter of *AUMO1* in the ChIP-seq data. The bar at the bottom represents the position of the DNA fragment containing the ACGTG(G/T)(C/A) motif which indicated with black dots. C, Verification of OsABF1V direct binding to the indicated DNA fragments as shown in B by ChIP-qPCR analysis. ChIP samples were prepared using anti-VP16 antibodies and subjected to qPCR analysis. Results of ChIP-qPCR were quantified by normalization of the immunoprecipitation signal with the corresponding input signal. The binding to 25S rDNA was used as a negative control. The means ± s.d. (n = 3) are shown. D, Verification of OsABF1 direct binding sites in the *AUMO1* promoter by ChIP-qPCR analysis. ChIP samples were prepared using WT rice and precipitated with anti-OsABF1 antibodies or with the pre-immune serum as a negative control. The means ± s.d. (n = 3) are shown.

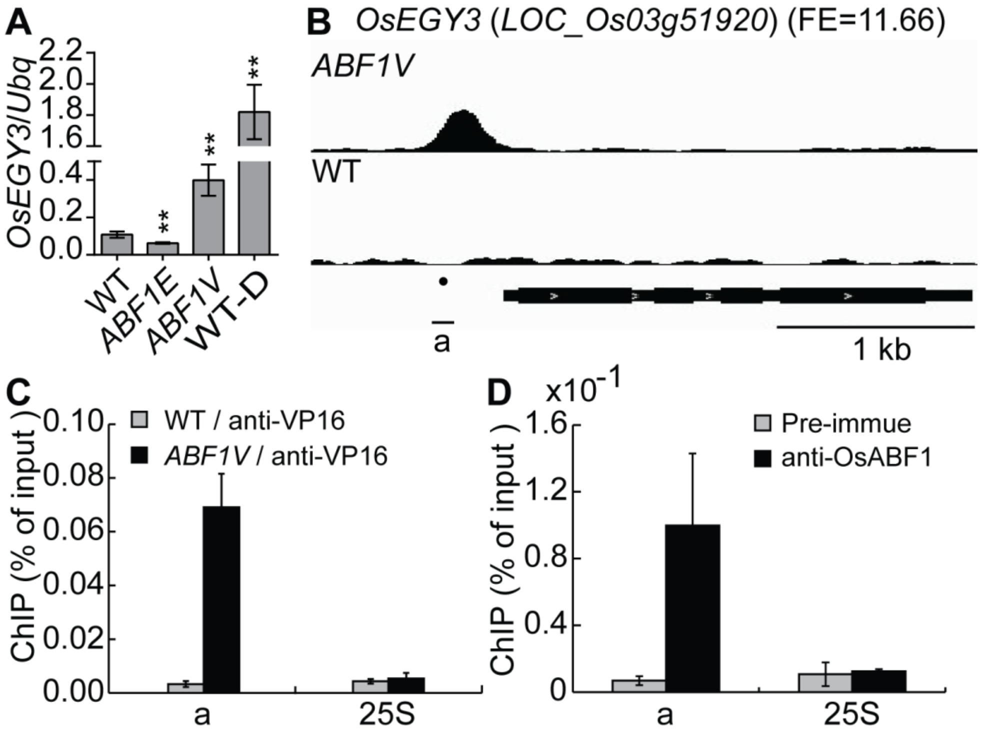

Supplemental Figure S8. Identification of *OsEGY3* as a direct target of OsABF1. A, Transcriptional analysis of *OsEGY3* in the WT, the *ABF1E* and *ABF1V* transgenic lines, and the WT-D by qRT-PCR with normalization to *Ubq*. Mean values ± s.e.m. are shown (Student's *t*-tests, $**p < 0.01$, n = 3). B, ABF1V binding profile in the promoter of *OsEGY3* in the ChIP-seq data. The bar at the bottom represents the position of the DNA fragment containing the ACGTG(G/T)(C/A) motif which indicated with black dots. C, Verification of OsABF1V direct binding to the indicated DNA fragment as shown in (B) by ChIP-qPCR analysis. ChIP samples were prepared using anti-VP16 antibodies and subjected to qPCR analysis. Results of ChIP-qPCR were quantified by normalization of the immunoprecipitation signal with the corresponding input signal. The binding to 25S rDNA was used as a negative control. The means ± s.d. (n = 3) are shown. D, Verification of OsABF1 direct binding sites in the *OsEGY3* promoter by ChIP-qPCR analysis. ChIP samples were prepared using WT rice and precipitated with anti-OsABF1 antibodies or with the pre-immune serum as a negative control. The means ± s.d. (n = 3) are shown.

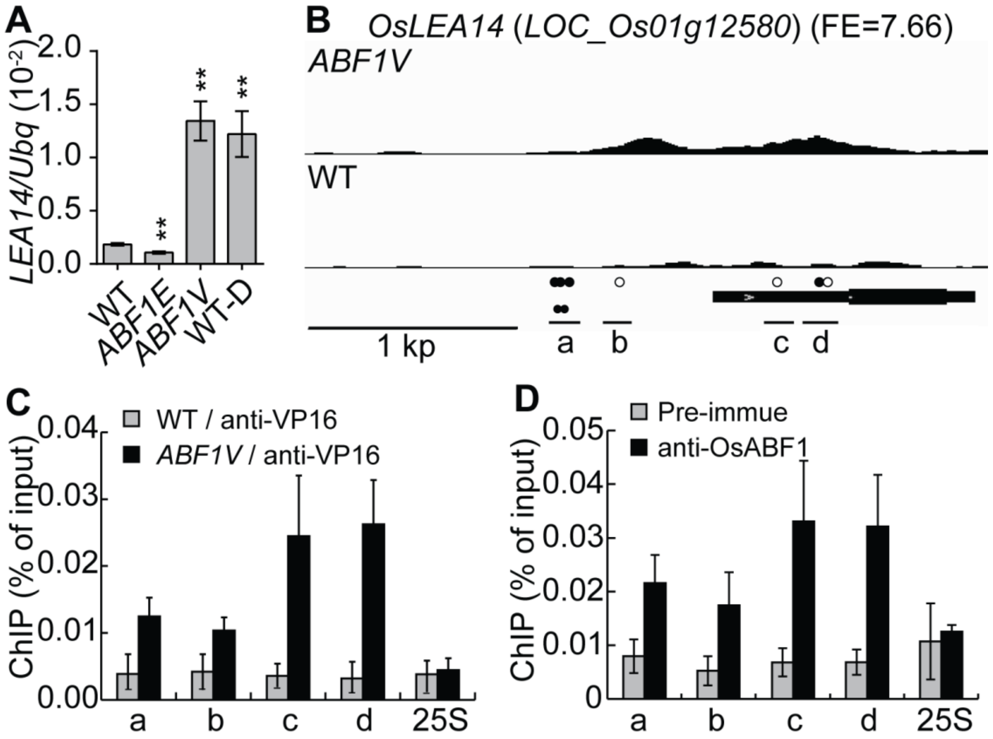

Supplemental Figure S9. Identification of *LEA14* as a direct target of OsABF1. A, Transcriptional analysis of *LEA14* in the WT, the *ABF1E* and *ABF1V* transgenic lines, and the WT-D by qRT-PCR with normalization to *Ubq*. Mean values ± s.e.m. are shown (Student's *t*-tests, $**p < 0.01$, n = 3). B, ABF1V binding profile in the promoter of *LEA14* in the ChIP-seq data. The bars at the bottom represent the distribution of DNA fragments containing the ACGTG(G/T)(C/A) motif or ACGT core as indicated by black or white dots. C, Verification of OsABF1V direct binding to the indicated DNA fragment as shown in B by ChIP-qPCR analysis. ChIP samples were prepared using anti-VP16 antibodies and subjected to qPCR analysis. Results of ChIP-qPCR were quantified by normalization of the immunoprecipitation signal with the corresponding input signal. The binding to 25S rDNA was used as a negative control. The means ± s.d. (n = 3) are shown. D, Verification of OsABF1 direct binding sites in the *LEA14* promoter by ChIP-qPCR analysis. ChIP samples were prepared using WT rice and precipitated with anti-OsABF1 antibodies or with the pre-immune serum as a negative control. The means ± s.d. (n = 3) are shown.

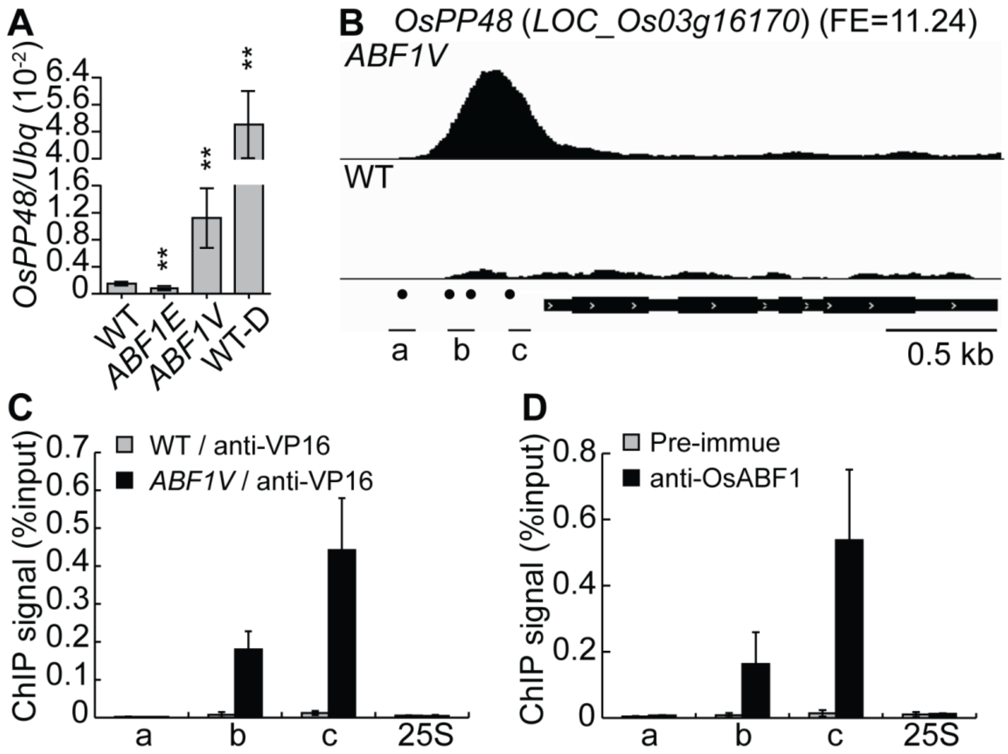

Supplemental Figure S10. Identification of *OsPP48* as a direct target of OsABF1. A, Transcriptional analysis of *OsPP48* in the WT, the *ABF1E* and *ABF1V* transgenic lines, and the WT-D by qRT-PCR with normalization to *Ubq*. Mean values ± s.e.m. are shown (Student's *t*-tests, ***p* < 0.01, n = 3). B, ABF1V binding profile in the promoter of *OsPP48* in the ChIP-seq data. The bar at the bottom represents the position of the DNA fragment containing the ACGTG(G/T)(C/A) motif which indicated with black dots. C, Verification of OsABF1V direct binding to the indicated DNA fragment as shown in B by ChIP-qPCR analysis. ChIP samples were prepared using anti-VP16 antibodies and subjected to qPCR analysis. Results of ChIP-qPCR were quantified by normalization of the immunoprecipitation signal with the corresponding input signal. The binding to 25S rDNA was used as a negative control. The means ± s.d. (n = 3) are shown. D, Verification of OsABF1 direct binding sites in the *OsPP48* promoter by ChIP-qPCR analysis. ChIP samples were prepared using WT rice and precipitated with anti-OsABF1 antibodies or with the pre-immune serum as a negative control. The means ± s.d. (n = 3) are shown.

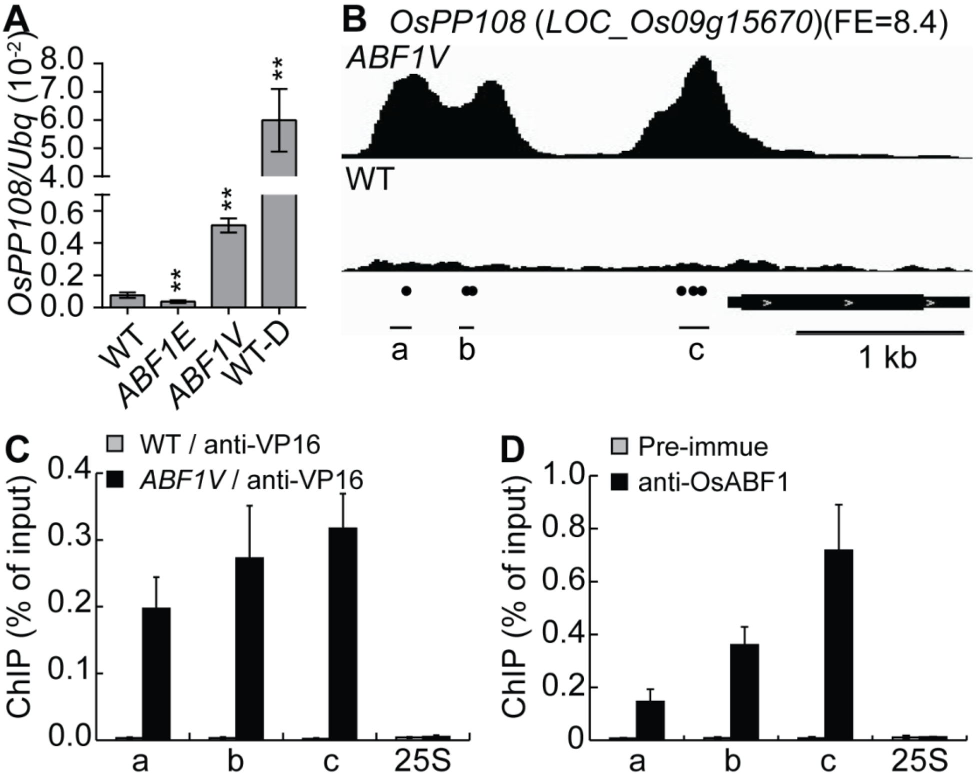

Supplemental Figure S11. Identification of *OsPP108* as a direct target of OsABF1. A, Transcriptional analysis of *OsPP108* in the WT, the *ABF1E* and *ABF1V* transgenic lines, and the WT-D by qRT-PCR with normalization to *Ubq*. Mean values ± s.e.m. are shown (Student's *t*-tests, $**p < 0.01$, n = 3). B, ABF1V binding profile in the promoter of *OsPP108* in the ChIP-seq data. The bar at the bottom represents the position of the DNA fragment containing the ACGTG(G/T)(C/A) motif which indicated with black dots. C, Verification of OsABF1V direct binding to the indicated DNA fragment as shown in B by ChIP-qPCR analysis. ChIP samples were prepared using anti-VP16 antibodies and subjected to qPCR analysis. Results of ChIP-qPCR were quantified by normalization of the immunoprecipitation signal with the corresponding input signal. The binding to 25S rDNA was used as a negative control. The means ± s.d. (n = 3) are shown. D, Verification of OsABF1 direct binding sites in the *OsPP108* promoter by ChIP-qPCR analysis. ChIP samples were prepared using WT rice and precipitated with anti-OsABF1 antibodies or with the pre-immune serum as a negative control. The means ± s.d. (n = 3) are shown.

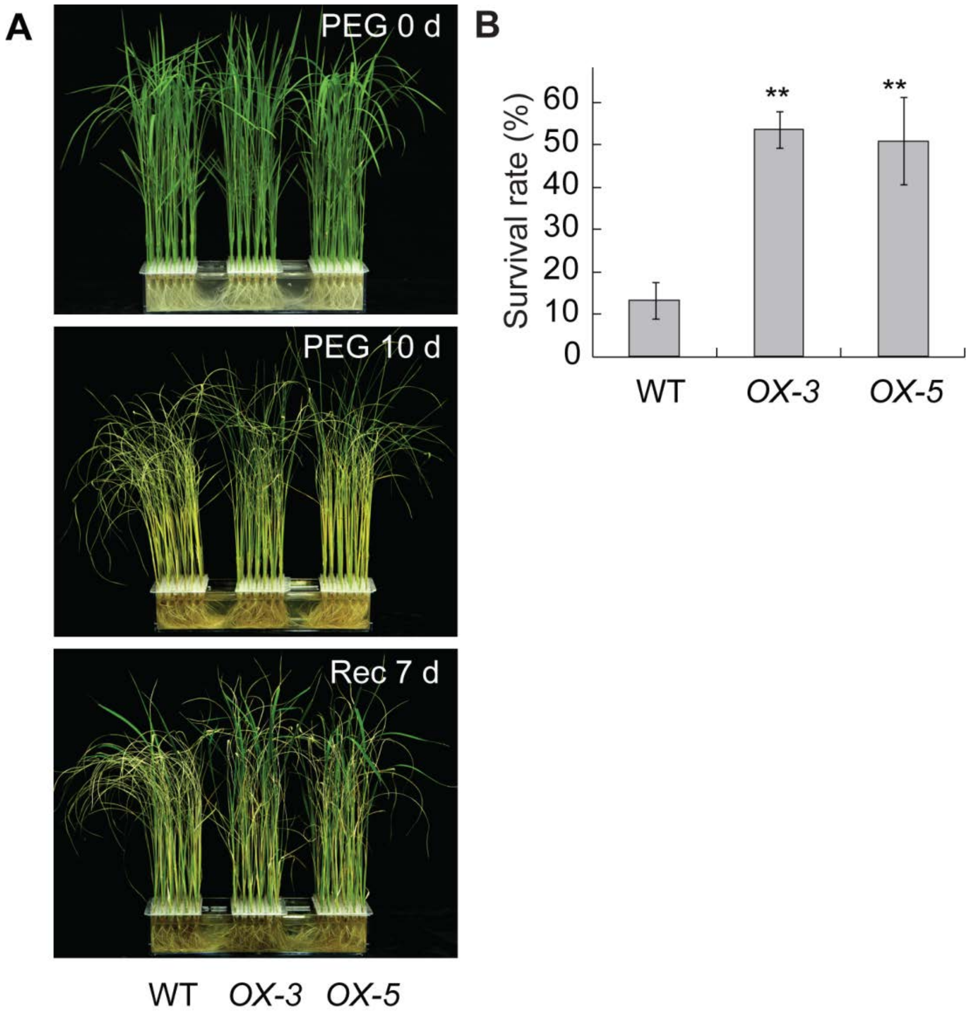

Supplemental Figure 12. Performance of *COR413-TM1* overexpression lines under osmotic stress. A, The images of WT and the *COR413-TM1* overexpression plants (*OX-3* and *OX-5*) subjected to PEG treatment. 21-day-old seedlings grown in water were transferred into 20% PEG for 10 days (PEG 10 d) and then recovered in water for 7 days (Rec 7 d). B, Survival rate of the indicated genotypes subjected to PEG treatment as in A. Mean values ± s.d. are shown. The value of the indicated genotype was compared to that of the WT (Student's *t*-tests, $^{**}p < 0.01$, n = 3).

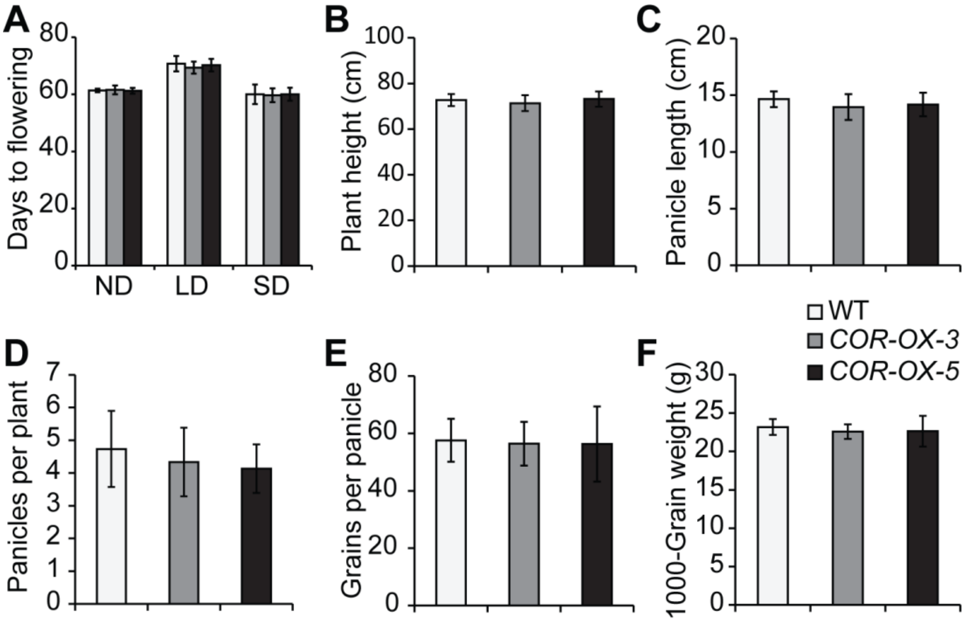

Supplemental Figure S13. Phenotype analysis of *COR413-TM1* overexpression plants.

(A) - (F): Comparison of heading date, plant height ,panicle length, panicle per plant, grains per panicle and 1000 grain weight between WT and *COR413-TM1* overexpression rice. Data are shown as means ± s.d. (Student's t tests, *P < 0.05, **P < 0.01, n=60).

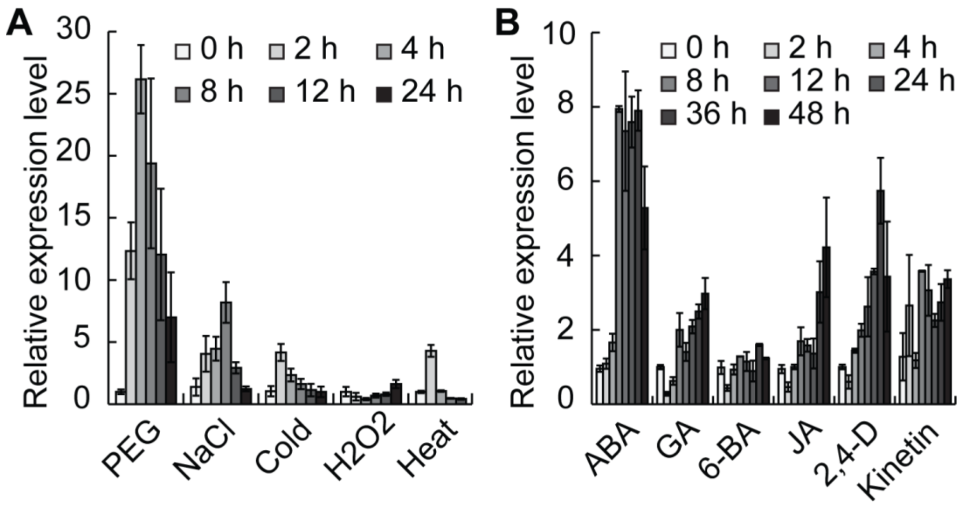

Supplemental Figure S14. Dynamic transcription of *OsCOR413-TM1* under abiotic stress or hormone treatments. A, Time-series mRNA levels under abiotic stress. B, Time-series mRNA levels under hormone treatments. The seedlings were grown in continuous light for 3 weeks and then subjected to the indicated treatments.

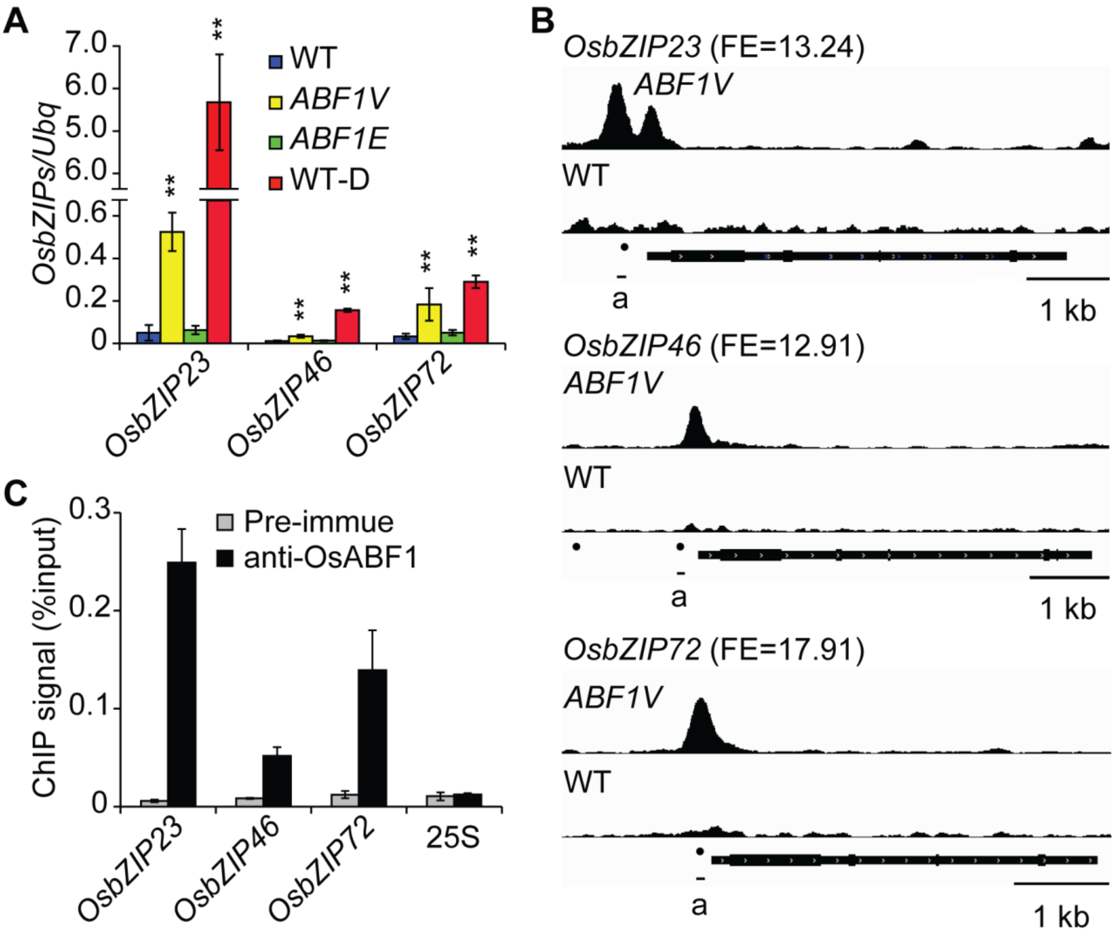

Supplemental Figure S15. Identification of *OsbZIP23*, *OsbZIP46*, and *OsbZIP72* as direct targets of OsABF1. A, Transcriptional analysis of *OsbZIPs* in the WT, the *ABF1E* and *ABF1V* transgenic lines, and the WT-D by qRT-PCR with normalization to *Ubq*. Mean values ± s.e.m. are shown (Student's *t*-tests, $^{**}p < 0.01$, n = 3). B, ABF1V binding profile in the promoter of *OsbZIPs* in the ChIP-seq data. The bar at the bottom represents the position of the DNA fragment containing the ACGTG(G/T)(C/A) motif which indicated with black dots. C, Verification of OsABF1 direct binding to the indicated DNA fragment "a" as shown in (B) by ChIP-qPCR analysis. ChIP samples were prepared using WT rice and precipitated with anti-OsABF1 antibodies or with the pre-immune serum as a negative control. The means ± s.d. (n = 3) are shown. Results of ChIP-qPCR were quantified by normalization of the immunoprecipitation signal with the corresponding input signal. The binding to 25S rDNA was used as a negative control. The means ± s.d. (n = 3) are shown.
